# Recursive music elucidates neural mechanisms supporting the generation and detection of melodic hierarchies

**DOI:** 10.1007/s00429-020-02105-7

**Published:** 2020-06-26

**Authors:** Mauricio J. D. Martins, Florian Ph. S. Fischmeister, Bruno Gingras, Roberta Bianco, Estela Puig-Waldmueller, Arno Villringer, W. Tecumseh Fitch, Roland Beisteiner

**Affiliations:** 1 Berlin School of Mind and Brain, Humboldt Universität zu Berlin, Berlin, Germany; 2 Max Planck Institute for Human Cognitive and Brain Sciences, Leipzig, Germany; 3 Clinic for Cognitive Neurology, University Hospital Leipzig, Leipzig, Germany; 4 Institut Jean Nicod, Département d’Etudes Cognitives, ENS, EHESS, CNRS, PSL Research University, Paris, France; 5 Institute of Psychology, University of Graz, Graz, Austria; 6 Department of Biomedical Imaging and Image-Guided Therapy, Medical University of Vienna, Vienna, Austria; 7 Institute of Psychology, University of Innsbruck, Innsbruck, Austria; 8 UCL Ear Institute, University College London, London, UK; 9 Department of Behavioral and Cognitive Biology, University of Vienna, Vienna, Austria; 10 Department of Neurology, High-Field MR Center of Excellence, Medical University of Vienna, Vienna, Austria

**Keywords:** IFG, Hippocampus, Recursion, Hierarchy, STG, Music

## Abstract

The ability to generate complex hierarchical structures is a crucial component of human cognition which can be expressed in the musical domain in the form of hierarchical melodic relations. The neural underpinnings of this ability have been investigated by comparing the perception of well-formed melodies with unexpected sequences of tones. However, these contrasts do not target specifically the representation of rules generating hierarchical structure. Here, we present a novel paradigm in which identical melodic sequences are generated in four steps, according to three different rules: The Recursive rule, generating new hierarchical levels at each step; The Iterative rule, adding tones within a fixed hierarchical level without generating new levels; and a control rule that simply repeats the third step. Using fMRI, we compared brain activity across these rules when participants are imagining the fourth step after listening to the third (generation phase), and when participants listened to a fourth step (test sound phase), either well-formed or a violation. We found that, in comparison with Repetition and Iteration, imagining the fourth step using the Recursive rule activated the superior temporal gyrus (STG). During the test sound phase, we found fronto-temporo-parietal activity and hippocampal de-activation when processing violations, but no differences between rules. STG activation during the generation phase suggests that generating new hierarchical levels from previous steps might rely on retrieving appropriate melodic hierarchy schemas. Previous findings highlighting the role of hippocampus and inferior frontal gyrus may reflect processing of unexpected melodic sequences, rather than hierarchy generation per se.

**Electronic supplementary material:**

The online version of this article (10.1007/s00429-020-02105-7) contains supplementary material, which is available to authorized users.

## Introduction

The human ability to represent and generate complex hierarchies is an intriguing phenomenon. Although some animal species seem able to represent simple hierarchies during social and spatial navigation (Buzsáki and Moser 2013; McKenzie et al. 2014; Seyfarth and Cheney 2014), human hierarchical cognition has both a larger scope and depth. First, humans can generate hierarchical structures in multiple domains including language, music, and complex action sequencing (Fitch and Martins 2014), and second, there is no limit, in principle, to the depth we can add to hierarchical structures (Hauser et al. 2002), except for those imposed by the limits of human working memory.

This generalized and unbounded generativity is supported by capacities for hierarchical embedding and recursion. Hierarchical embedding is a process through which an element, or set of elements, is made ‘subordinate’ to another ‘dominant’ element. For instance, in English, when the word ‘film’ is embedded in ‘committee’ to form [[film] committee], it refers to a kind of committee, not a kind of film. Recursion is the process through which a function’s output is used again as input to the same function. For instance, the natural numbers are described by the recursive function *N*_i_ = *N*_*i*−1_ + 1, which generates the infinite set {1, 2, 3,…}. By combining these two properties—recursion and hierarchical embedding—we can generate hierarchies of unbounded depth. For instance, by using the recursive embedding rule NP → [[NP] NP] we can add ‘student’ to ‘film committee’ and obtain [[[student] film] committee] and so on.

The ability to use recursive hierarchical embedding (RHE) has been demonstrated in the domains of language (Perfors et al. 2010), music (Martins et al. 2017), vision (Martins et al. 2014a, b, 2015) and in the motor domain (Martins et al. 2019). While behavioural research suggests that RHE is instantiated by similar cognitive resources across these domains (Martins et al. 2017), it is not clear to what extent it is also supported by similar neural mechanisms. In previous research we have investigated the neural implementation of RHE in the visual and motor domains (Martins et al. 2014a, 2019). Here, we will use music-like stimuli to extend this research to the auditory domain.

Within the auditory domain, previous research has focused on musical harmonic syntax, which describes a set of rules governing hierarchical tonal relations between notes and chords (Lerdahl and Jackendoff 1977). These relations are learned through processes of music enculturation and thus create expectations which, when violated, cause certain sequences to be perceived as incorrect or surprising, similar to the effects of grammatical violations in language (Beisteiner et al. 1999; Rohrmeier and Koelsch 2012; Rohrmeier et al. 2015; Tillmann 2012). Violations of music syntax consistently activate the inferior frontal gyrus (IFG) and the superior temporal gyrus (STG) (Bianco et al. 2016; Koelsch et al. 2002, 2005; rev. Salimpoor et al. 2015; Seger et al. 2013). Moreover, these areas are also active when contrasting melodies vs unstructured tones (Minati et al. 2008).

The specific roles of IFG and STG in the processing of hierarchical structures are unclear. Because IFG is also involved in processing syntax in language and in action, this area has been thought as essential to the processing of hierarchies in general (Fadiga et al. 2009; Fazio et al. 2009; Fitch and Martins 2014; Maess et al. 2001; Musso et al. 2015; Patel 2003). Shared activation patterns between language and music have been dubbed syntactic integration resource hypothesis (SSIRH) (Patel 2003), and since then several neuroimaging studies have highlighted these commonalities (reviewed in Peretz et al. 2015). However, it has been pointed out that, because the contrasted stimuli have different surface characteristics (correct vs incorrect, local vs. long-distance dependencies), these similar patterns of activity may reflect domain-general resources such as working memory or cognitive control, rather than specifically reflecting increased structural load in the combinatorial activities used to generate hierarchies (Bigand et al. 2014; Novick et al. 2005; Patel and Morgan 2017; Rogalsky et al. 2011).

STG has also been found to be active during the processing of music and linguistic syntax (Sammler et al. 2013), and during the processing of both lyrics and tones (Sammler et al. 2010). This region seems to be generally active in the processing of auditory stimuli, but it also stores tonal maps (Rogalsky et al. 2011) and schemas of sound events (Lee et al. 2011), and is active in music imagery and familiarity for structural relations (Herholz et al. 2012). This evidence for structure sensitivity suggests that processing music is biased by expectations based on such stored schemas (reviewed in Salimpoor et al. 2015). Furthermore, STG activations seem to differ between local and global violations of music syntax, with the former generating bilateral, and the latter left-lateralized, activations (Stewart et al. 2008). This inter-level segregation is an essential building block for the processing of hierarchies, since the understanding of hierarchy necessitates the ability to represent different levels of structural organization.

In addition to these areas, recent research has highlighted the role of the hippocampus in the processing of hierarchical structures across a variety of domains (Garvert et al. 2017; McKenzie et al. 2014; Schapiro et al. 2013; Stachenfeld et al. 2017). The hippocampus is generally involved in the formation of schemas through memory generalization processes (Berens and Bird 2017). When multiple experiences with similar features occur, these can form context-independent memory structures. These memory structures can contain schema nodes activated in a hierarchical fashion navigating between local and global features of the stimuli (Cooper and Shallice 2006; Stachenfeld et al. 2017). In the musical domain, hippocampus activity increases with music expertise while processing musical syntax (Groussard et al. 2010), similarly to STG (Groussard et al. 2010; Koelsch et al. 2005). This suggests that both areas may be important for the formation and retrieval of tonal schemas. Furthermore, when the recognition of previously learned music structures is measured, instead of violation processing, hippocampus activity increases with familiarity while IFG activity decreases (Watanabe et al. 2008).

Summarizing, previous studies leave unclear to what extent current experimental paradigms specifically isolate hierarchical generativity or, more specifically, recursive hierarchical embedding. As noted above, studies investigating the processing of music syntax usually rely on the contrast between violations vs. well-formed tone (or chord) sequences, or melodies vs. scrambled tones. Because different stimuli are contrasted, brain activity in these studies could reflect general working memory, cognitive control, or other processes involved while parsing superficial features of these stimuli. This makes it difficult to isolate the mechanisms supporting the representation of the underlying hierarchical structure using established paradigms.

In this fMRI experiment, we introduce a novel paradigm which partially reproduces features of previous studies but allows us to isolate the cognitive processes underlying internal representations of hierarchical tone sequences. Here, we repeatedly apply RHE rules to musical tones, forming auditory ‘fractal’ hierarchies in discrete steps. ‘Fractal’ refers to the structural similarity across hierarchical levels that results from applying a recursive rule. Well-trained participants listen to the first three steps, each of which generates a new level of the hierarchy. They then listen to a fourth step, and are asked to determine whether the new tone sequence containing an additional hierarchical level is consistent with the previous three steps.

We contrasted accuracy and brain activity associated with the ‘Recursive’ rule with a simpler ‘Iterative’ rule, which followed the same stepwise procedure. However, in Iteration, each step added elements within the same single level of the hierarchy, without generating new levels. Behavioural studies have shown that accuracy in using the Recursive rule in the music domain correlates with the same ability in the visual and action sequencing domains (Martins et al. 2017). Importantly, these shared capacities dissociated from those seen in Iteration. Our current procedure also included a control Repetition condition, in which participants were given a certain melodic structure in step three, and then asked whether step four was identical to step three or not. Crucially, the stimuli were identical across all rules in the fourth step. Hence, the stimuli to be imagined and heard were the same. This aspect of our design eliminates the potential confound of stimuli being perceptually different, isolating how identical stimuli were internally represented (as hierarchical vs. iterative).

Finally, we divided our neural activity analysis to contrast between two processing periods: (i) the generation phase, in a silent period between the third and fourth steps, in which participants use the rule parameters to imagine the tone sequence corresponding to the fourth step, and (ii) the test sound phase (the fourth step), in which participants listen to a tone sequence that is either the correct continuation of the third step, or a violation. This analysis allows us to separate the activity related to the internal generative act from the activity related to external stimulus processing. By separating these phases, we evaluated whether any of the brain regions discussed above is specifically involved in the generative act versus playing a more general role in the processing of melodic structures. We performed both whole brain analyses and ROI analyses targeting IFG, hippocampus and STG in both hemispheres, the most likely candidates to support generation of hierarchical structures, based on previous research.

## Methods

### Participants

Fifteen healthy participants (seven males and eight females, age range 20–35, *M* = 25.5) took part in the study. All participants were non-musicians: None had more than 2 years of music training and none practiced regularly with a musical instrument. All had normal or corrected-to-normal vision and audition, no history of neurological or psychiatric disease and no current use of psychoactive medications. All completed a short questionnaire screening for previous clinical history and a paper-and-pencil version of Raven’s progressive matrices (a test of non-verbal intelligence) (Raven et al. 1998) and the Melodic Memory Task from the Gold-MSI test battery (Müllensiefen et al. 2014). Participants were recruited online and most were university students. All participants were right-handed German native speakers. Participants gave informed written consent before the experiment in accordance with guidelines of the local ethics committee. Before the functional magnetic resonance imaging (fMRI) session, each participant was explicitly debriefed about both generating rules and practiced one or two blocks of the experimental task (with different stimuli) after which s/he received feedback. Participants were paid 30 Euros for their participation. The overall procedure comprised one hour of practice plus cognitive testing and approximately one and a half hours of fMRI scanning.

### Stimuli and behavioral tasks

**Fig. 1:**
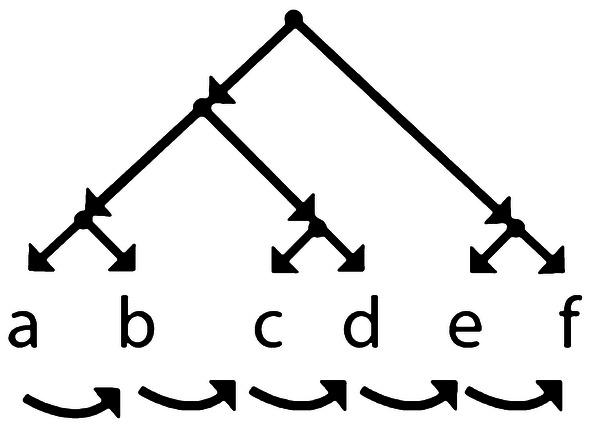
A collection of items (**a**–**f**) unfolding in time can be represented simultaneously as a sequence (bottom arrows) and a cognitive hierarchy (top structure)

The central cognitive construct that we aim to isolate is the capacity to represent rules which enable the generation of hierarchical structures. In previous work, we defined the distinct concepts of hierarchy and sequence using a graph theoretical framework (Udden et al. 2019): Sequence is a rooted directed acyclical graph (DAG) in which no node has more than one child, thus being limited to a single order along the root-to-terminal axis and to a single terminal (Fig. 1, bottom). On the other hand, a Hierarchy is a rooted DAG in which at least one node has more than one child, thus forming a branching tree (Fig. 1, top). The terminals (Fig. 1a–f) of a hierarchical structure are unordered (top) unless a sequence is imposed upon them (bottom). When this is the case, we obtain two distinct ordering structures: (1) a sequential terminal-to-terminal (horizontal) ordering, which in music corresponds to items unfolding in time; and (2) a hierarchical root-to-terminal (vertical) ordering, which in music can correspond to either a tonal or harmonic intervallic structure.

Following the conceptual framework, we designed a set of tasks described in detail in Martins et al. (2017), which we use in the current study. In this earlier study, we showed that both musicians and non-musicians can acquire recursive rules in the domain of melodic hierarchies and that this capacity (when contrasted with simple iteration) is predicted by the ability to understand recursion in the visual domain and with the capacity to solve the Tower of Hanoi, a recursive planning task.

**Fig. 2:**
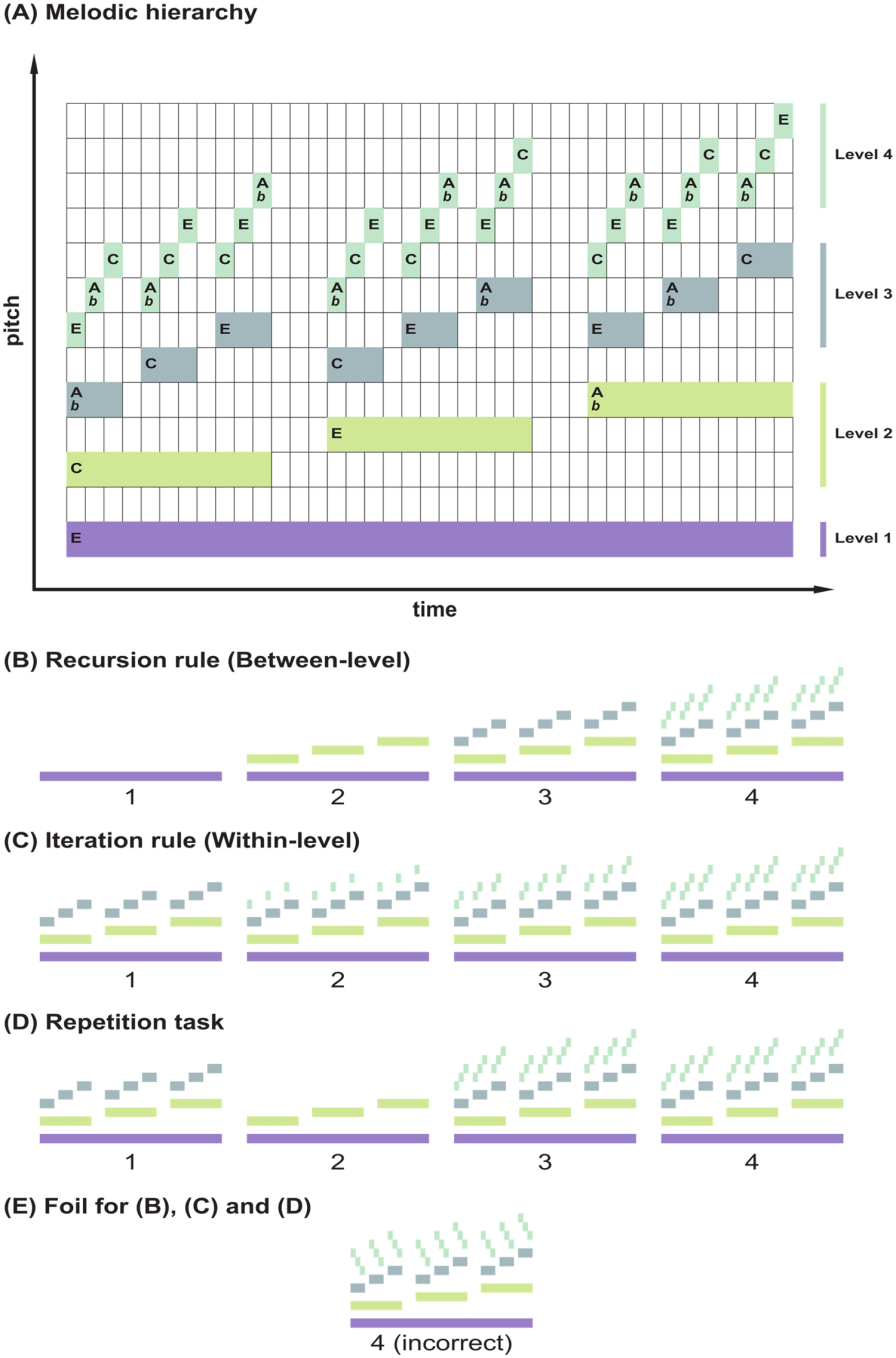
**a** Melodic hierarchical sequences. Colored items denote musical notes of a particular pitch and duration. Letters within these items denote the musical note (E, C and Ab) and the color denote their hierarchical level (1, 2, 3 or 4). Items within dominant (lower frequency) levels were of a longer duration than items in subordinate levels. Each item in Levels 1, 2 and 3 was dominant over a set of three other items of a higher pitch and with a certain melodic contour (in this figure ‘ascending’, see text for details). The pitch of a dominant item determined the pitches of the subordinate set according to pitch relations (major third or minor sixth) that were consistent across different levels. The melodic relations within each set of three and their contour were also consistent across levels. These sequences could be generated using: **b** Recursive rules, which added new hierarchical levels at each application step (1, 2, 3 and 4) or **c** Iterative rules, which added elements within a fixed level, without generating a new level. **d** a Repetition rule was also run, in which the first two steps were unrelated and then participants were asked whether step 4 was a repetition of step 3. Our test stimuli in the MR-scanner were the first four steps resulting from the application of these rules (or unrelated tone sequences in Repetition) plus an incorrect 4th step (**e**), which was used as a foil. **e** These foils were generated by applying a rule to generate the 4th step which was different from the rule used to generate the previous 3 steps (see Fig. 4 for details)

Our stimuli are based on the properties of melodic fractals (Fig. 2a). Melodic fractals are structures with several hierarchical levels in which the hierarchical relations between dominant and subordinate elements are kept the same across levels. Here, the elements of different levels are identified by their pitch and duration, with lower pitch and long duration elements being dominant over elements with higher pitch and shorter duration (Martins et al. 2017; Tamir-Ostrover and Eitan 2015).

Each note at level *n*, (*x*)_*n*_, is dominant over a sequence of subordinate tones at level *n* + 1: [(*x*1)_*n*+1_, (*x*2)_*n*+1_, (*x*3)_*n*+1_]. The generative rule determining the relationship between hierarchical levels includes four variables: inter-level interval, inter-tone interval, melodic contour and tone duration.

First, the inter-level interval *l* is the pitch interval between a dominant tone (*x*)_*n*_ and the subordinate with the lowest pitch (*x*1)_*n*+1_. *l* can be either 4 or 8 semitones (*l* ϵ {4,8}) and is constant across levels. We chose these particular musical intervals to avoid dissonance, because the new levels were added cumulatively and were played back simultaneously with dominant level tones (see Martins et al. 2017 for details).

Second, the inter-tone distance *t* is the pitch interval between each pair of adjacent items in the subordinate sequence. The distance *t* can be either 4 semitones (major third) or 8 semitones (minor sixth) (*t* ϵ {4,8}) and is constant across levels. Since the reference note within each triplet is (*x*2)_*n*+1_, we can write (*x*2)_*n*+1_ as *x*_*n*_ + *l* + *t*, or for simplicity (*x*2)_*n*+1_ = *x*_*n*_ + ϕ. Putting together the relationships between and within level, we obtain the generative rule: (*x*)_*n*_ → [(*x*)_*n*_ [(*x* − *t* + ϕ)_*n*+1_, (*x* + ϕ)_*n*+1_, (*x* + *t* + ϕ)_*n*+1_]], in which the dominant level (*x*)_*n*_ remains present, in addition to the new subordinate sequence [(*x* − *t* + ϕ)_*n*+1_, (*x* + ϕ)_*n*+1_, (*x* + *t* + ϕ)_*n*+1_].

Third, the subordinate sequence can have either an ascending or descending contour *c* ϵ {− 1, 1} (ascending = 1 or descending = − 1). Adding this parameter to the generative rule, we obtain the recursive hierarchical embedding rule:

$$(x)_{n} \to \left[ {\left( x \right)_{n} \left[ {(x - tc + \phi )_{n + 1} ,(x + \phi )_{n + 1} ,(x + tc + \phi )_{n + 1} } \right]} \right].$$

Finally, the duration of the (*x*)_*n*+1_ tones comprising the subordinate sequence are approximately 1/3 of the duration of the dominant (*x*)_*n*_ tone. We added short silent pauses between tones to facilitate discrimination of each individual tone. Pauses between clusters of three tones were longer than pauses within each cluster, which facilitated the perceptual separation between clusters. This resulted in a sound with a total duration of 7.4 s. For more details see Martins et al. (2017).

In addition to contour, inter- and intra-level intervals, which gave us 2 × 2 × 2 = 8 different fractal stimuli, there were four different starting tones for each stimulus, resulting in a pool of 8 × 4 = 32 different fractals.

As follows from above, the ‘Recursive rule’ (Fig. 2b) generated the final structure (Fig. 2a) in four steps, each step adding a new hierarchical level consistent with the previous. In contrast, the ‘Iterative rule’, which also generated the same structures in 4 steps, simply added new elements within a fixed hierarchical level, without generating new levels (Fig. 2c). The contrast between these rules thus taps into the representation of recursive processes that specifically generate several levels with a single rule. For each melodic hierarchy, in addition to a correct 4^th^ step, we generated an incorrect 4th step or foil (Fig. 2d). This foil was built by violating the rule contour parameter *c* from the third to the fourth step (“positional” foil), i.e. changing from ascending to descending melodic contour or vice versa. For each condition, we presented well-formed stimuli in half of the trials and foils in the other half.

Our third control condition was the ‘Repetition rule’, in which participants were first exposed to three unrelated melodic hierarchies and then asked to determine whether the 4th step was an exact repetition of the 3th step, or a different melodic hierarchy.

Two aspects are important in our design. First, our generative rule (*x*)_*n*_ → [(*x*)_*n*_ [(*x* − *tc* + ϕ)_*n*+1_, (*x* + ϕ)_*n*+1_, (*x* + *tc* + ϕ)_*n*+1_]] creates both a melodic sequence [(*x—tc* + ϕ)_*n*+1_, (*x* + ϕ)_*n*+1_, (*x* + *tc* + ϕ)_*n*+1_] and a sequence of harmonic intervals (of the tones in the same sequence in relation to the baseline tone (x)_*n*_). Presumably, participants could use either or both aspects to infer the rule. We ensured that the baseline tones (*x*)_*n*_ were perceptually available in the stimulus to facilitate the detection of the relationship between two hierarchical levels while reducing short term memory demands. While we cannot determine whether participants focused on the sequence of increasing/decreasing harmonic intervals or on the ascending/descending melodic sequence when listening to the stimuli, both perceptual experiences correspond to the same underlying rule, and are formally equivalent.

Second, in the 4th iteration, it is possible that participants paid attention only to the last two levels without tracking the full vertical harmonic structure. Importantly, before hearing the complete test stimuli with all levels played at once in the 4th iteration, they heard each level being introduced individually, step-by-step, in a 4-step procedure. In order to decode the hierarchical structure of the stimuli, and form correct expectations, they thus had to represent the generating rule binding the two highest levels of each step/iteration (and understand that the generating rule was consistent with that binding the previously heard levels). Thus, participants who were able to correctly identify continuations in the Recursive task needed to cognitively represent levels of the hierarchy, based on the formal hierarchical definition adopted here (see Udden et al. 2019), whether or not they perceptually attended to them in the final stimulus. Second, it is important to note that participants’ ability to represent this binding rule correlated strongly and specifically with similar abilities in the visual and motor domains (Martins et al. 2017), supporting the hypothesis that the Recursive task employed here isolates some aspects of hierarchical generativity.

### fMRI procedure

**Fig. 3:**
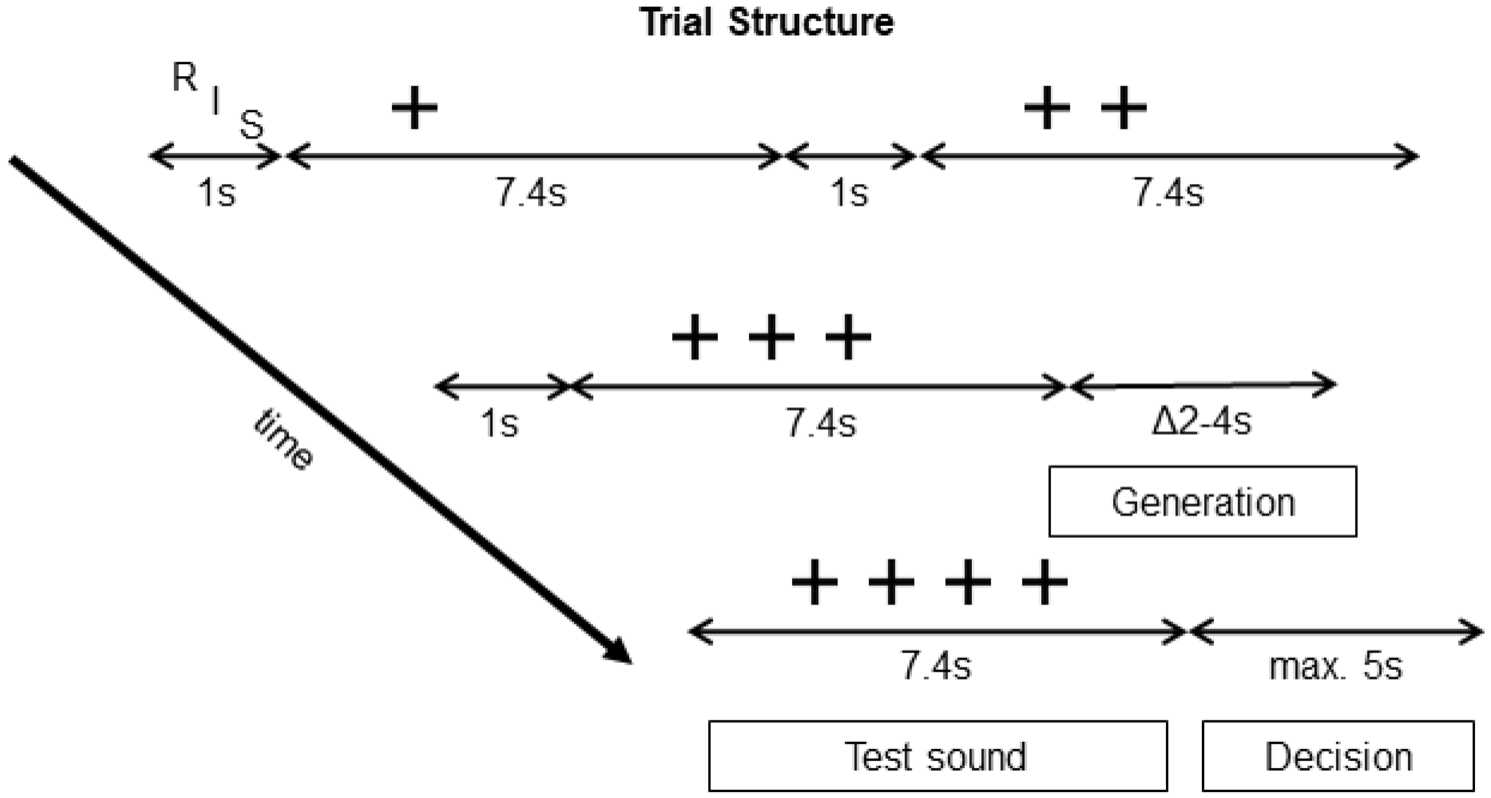
Trial structure. Inside the scanner, participants performed 4 sessions of 18 trials each [6 trials of Recursion (R), 6 of Iteration (I) and 6 of Repetition (S)]. All trials were constructed as the following: First, there was a letter indicating the category of the trial (F, I, R), then the first three steps were played while crosshairs were presented on the screen. After the first three steps were presented, participants had a period of 2–4 s to imagine how the 4th step would sound like (generation phase). Then they were presented with the test sound sequence (test sound phase), which could be either a correct or an incorrect 4th step. They were asked to decide whether the tone sequence was correct or incorrect and to present their choice by pressing one of two buttons in a button box (decision phase, which ran until a button press up to a maximum of 5 s). In addition, we separated the test sound phase in two equal parts and focused our analysis in the first part, where potential violations could already be detected (see methods for details). By doing so we could model activations related to preparation for response and to potential attention drifts. *max.* maximum

Before each trial (Fig. 3) participants were shown a letter indicating the trial rule [Recursion (R), Iteration (I) or Repetition (S)]. Then they listened sequentially to the first 3 steps resulting from the application of the rule (or three unrelated hierarchies in ‘Repetition’). Each step was accompanied by 1, 2, or 3 crosshairs on the screen indicating the corresponding step. After the 3rd step there was a generation phase, ranging between 2 and 4 s in which participants were asked to imagine how the 4th step would sound like. Then, in the test sound phase, participants were asked to listen to the test sound, and to determine whether this tone sequence was a correct 4th step or a foil. They delivered the response in the decision phase after the test phase by pressing a button on a button box (using LEFT thumb if it was correct and RIGHT thumb if it was incorrect).

Crucially, the same final test sound sequences were used in the different task rules (Recursion, Iteration or Repetition). Thus, cognitive differences in the generation and test phases would relate to how identical test sound sequences were generated and represented. Importantly, in order to account for potential differences in the BOLD signal due to the first three steps, we included these steps in the first level fMRI analysis.

Each participant performed 4 sessions of 18 trials each with intermixed conditions [6 trials of Recursion (R), 6 of Iteration (I) and 6 of Repetition (S)]. Half of the trials presented a correct 4th step and half presented a Foil. The set of six trials of each ‘correctness’ category was further divided in three stimuli with ascending contour, and three stimuli with descending contour. The number of stimuli of each inter-level (IL) and inter-tone (IT) intervals (8 vs. 4) was balanced for each rule across the full 4-session set of trials (4 × 6 = 24): 12 stimuli (6 correct and 6 foils) of IT/IL interval 8 and 12 stimuli of IT/IL interval 4. Session order was pseudorandomized across participants. Optimal trial sequence and Jittering parameters in the planning phase were obtained using Optseq2 (Greve 2002).

### Pretesting

**Fig. 4:**
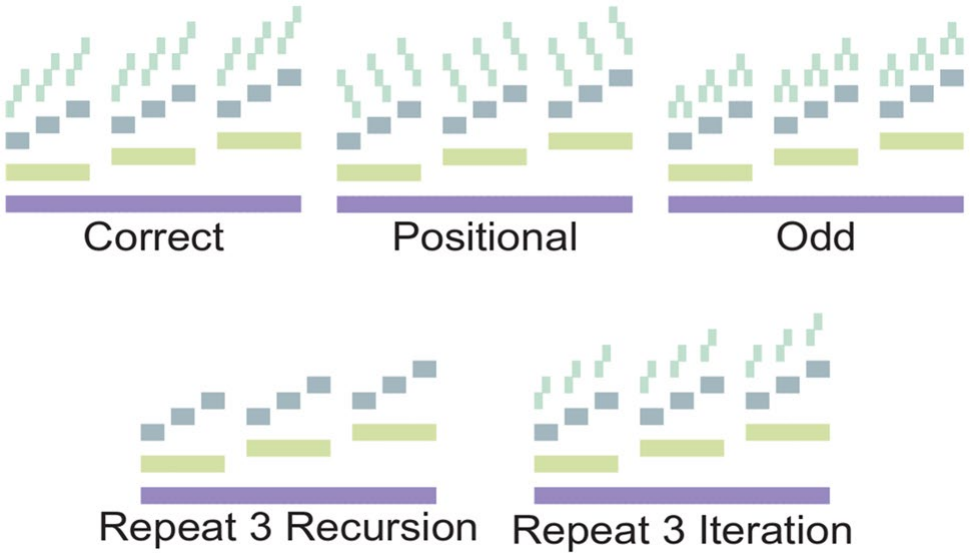
Correct 4th step and foil types in the first and second pre-testing sessions. The first pre-training session was a 2-forced choice discrimination task, while the second was a 1-forced choice detection task. During both sessions, we used three foil categories (Positional, Odd and Repeat) similarly to Martins et al. (2017)) to prevent participants from developing simple auditory heuristic strategies and to incentivize participants to imagine the test sound sequence during the generation phase. However, in the fMRI experiment we used only the ‘Position’ foils to homogenize stimuli across conditions and facilitate analysis (The Repeat foil was different between Iteration and Recursion, and the Odd foil is a salient contour which is easy to detect)

No more than one week before the MRI testing, participants performed a 2-h pretesting session. In this session, participants were explicitly and verbally instructed about the task rules, aided by slides shown on a screen (Powerpoint presentation and sounds available as Supplementary Materials). Then we familiarized participants with the stimuli and assessed whether their performance was adequate. In this session, participants were explicitly instructed about the recursive and iterative rules and then performed 2-forced choice “discrimination” tasks with 12 Recursion and 12 Iteration trials (described in detail in (Martins et al. 2017)). These discrimination tasks were similar to the procedure described above, except that two-tone sequences were consecutively presented (in a random order) in the test sound phase instead of one. One of the test sound sequences was a correct (step 4) continuation and the other was a foil. Participants had to indicate which was correct by selecting the appropriate box in the screen using the mouse. In the pretesting phase, we used two additional foil categories in relation to the fMRI procedure: (1) A ‘Repeat 3’ foil, which is simply a repetition of the third step; and (2) an “Odd foil” in which one element in each set of three tones was misplaced in the subordinate level (Fig. 4). Participants performed a maximum of two runs with each task, until their performance was at least 8/12 correct.

Then, on the scanning day, we performed one warm-up session with the same experimental procedure used in the MR, but outside the scanner. In this session, we used all three foil categories: (1) Repetition, (2) Odd, and (3) Positional. We kept the three foils in this phase to prevent participants from developing simple auditory heuristics based on the detection of feature specific to ‘Positional foils’, thus incentivizing participants to try to imagine the correct 4th step before the test sound phase. However, in the final sessions in scanner we only used the ‘Positional’ foils so that we could present exactly the same test sound stimuli in the Recursion, Iteration and Repetition trials, thus facilitating analysis and interpretation.

In addition to the pretesting familiarization phase, participants’ melodic memory was assessed using the Melodic Memory Task from the Gold-MSI test battery (Müllensiefen et al. 2014). For the latter, to ensure normal musical perception abilities, participants were asked to listen to pairs of short melodies (containing between 10 and 17 notes) and to indicate whether the two melodies had an identical pitch interval structure or not (by selecting “same” or “different”). In “same” trials, the second melody had the same pitch interval structure as the first one, but was transposed by a semitone or by a fifth. In “different” trials, in addition to being transposed, the second melody was modified by changing two notes by an interval varying between 1 and 4 semitones (for details, see Mullensiefen et al. 2014). The task was composed of 13 trials, including 2 initial training trials, and had a total duration of around 10 min. Percentage correct was calculated and all participants scored within 2 standard deviations of the norm for the UK (Müllensiefen et al. 2014).

### fMRI data acquisition

Functional and anatomical data were acquired with a 3 T TIM Trio system (Siemens, Erlangen, Germany) using a 32-channel Siemens head coil. For the functional magnetic resonance images (fMRI) an optimized 2D single-shot echo planar imaging (EPI) sequence with TR 2000 ms and TE 32 ms was used. Altogether, in four sessions of 420 volumes each, functional images were acquired with FOV of 210 × 210 mm, in-plane matrix 90 × 90, with 36 slices of 2.7 mm thickness and 20% gap (voxel size 2.3 mm × 2.3 mm × 2.7 mm) aligned parallel to the AC-PC plane, and a flip angle of 73°. The total acquisition time was 56 min. Additionally, anatomical high-resolution T1-weighted MR images were collected using a 3D MPRAGE sequence (TE = 3.02 ms, TR = 2190 ms, inversion time [TI] = 1300 ms) with a matrix size of 250 × 250 × 256, with isometric voxels with a nominal side length of 0.9 mm, flip angle of 9° and GRAPPA acceleration factor 2.

### fMRI data preprocessing

fMRI data of 15 participants were analysed with statistical parametric mapping (SPM8; Welcome Trust Centre for Neuroimaging; https://www.fil.ion.ucl.ac.uk/spm/software/spm8/). Functional data were pre-processed by following standard spatial pre-processing procedures. They consisted of: slice time correction (by means of cubic spline interpolation method), spatial realignment and co-registration of functional and anatomical data. Then, we performed a classical spatial normalisation into the MNI (Montreal Neurological Institute) stereotactic space that included resampling to 2 × 2 × 2 mm voxel size. Finally, data were spatially low-pass filtered using a 3D Gaussian kernel with full-width at half-maximum (FWHM) of 8 mm.

For single-subject analyses, evoked hemodynamic responses for the different event types were modelled within a comprehensive general linear model (GLM). This first level model included the generation phase, the test sound phase and the decision phase (Fig. 3). We also included an event comprising the period between the beginning of the trial and the end of step 3 (the prior phase). With the inclusion of this prior phase in the first level analysis we modelled the BOLD differences between trial types (Recursion, Iteration and Repetition) within steps 1, 2 and 3, and sought to extract these effects from the generation and test sound phases.

To summarize, the first level GLM included: (1) the prior phase, which was the period between trial onset and the end of step 3, with duration *d* = 26.04 s; (2) the generation phase, with onset 2–4 s prior to the test sound (step 4), and with *d* = 2–4 s; (3) the test sound phase, *d* = 7.4 s, corresponding to step 4; and (4) the decision phase, comprising the period between the end of step 4 and the response button press. We further divided the test sound phase (composed of 3 clusters of 9 tones each) in two halves, the first comprising the first cluster (*d* = 2.4 s) and the second comprising the remaining duration of the tone sequence. The rationale behind this division was the following: because the auditory stimuli were organized in 3 clusters with identical structure (Fig. 2a), it was possible to detect violations to well-formed tone sequences within the first cluster (Fig. 2e). In order to model potential attention drifts or other artifacts related to motor response preparation in the later phases of the test sound phase, both parts of the latter phase were included in the first level GLM. For the second-level analysis only the first part of the test sound phase was included in the GLM. For comparison, results showing the full test sound phase of 7.4 s are depicted in Supplementary Materials and are identical, except with greater activity in the motor cortex areas, lateralized according to the response button: LEFT for incorrect and RIGHT for correct.

To this design, we added estimated motion realignment parameters as covariates of no interest to regress out residual motion artefacts and increase statistical sensitivity. In addition, a 128 s cutoff high-pass filter was applied to account for low-frequency drifts and signal fluctuations.

Responses corresponding to the generation [RULE: Recursion (R), Iteration (I) and Repetition (S)], the test sound [RULE × CORRECTNESS: Correct (Co) vs. Foil (Fo)], and the decision (BUTTON PRESS: left vs. right) phases were then summarized across the four sessions and entered into a second-level GLM.

### fMRI statistical analysis

Group analyses were conducted in the context of the general linear model (GLM) separately for the generation and test sound phases. For these analyses, flexible factorial within-subject ANOVAs were performed with the factor RULE (Recursion, Iteration and Repetition); for the test sound phase analyses CORRECTNESS (Correct vs. Foil) was added as additional factor. All analyses were restricted to grey matter, i.e. individual whole brain data were masked using a voxel wise global gray matter threshold of 0.25, and finally thresholded to FWE *p*s > 0.05 for significance. We also modelled button press in the decision phase.

Within these models further statistical parametric maps using t contrasts were constructed to disentangle significant main effects. In the generation phase, t contrasts were calculated between each RULE. In the test sound phase, t contrasts were calculated between RULE and between Correct and Foil sounds.

We controlled family-wise error rate (FWER) of clusters below 0.05 with a cluster-forming height-threshold of 0.001. Anatomical labels are based on Harvard–Oxford cortical structural atlas implemented in FSL (https://fsl.fmrib.ox.ac.uk/fsl/fslwiki/Atlases).

### Region of interest (ROI) analyses

**Fig. 5:**
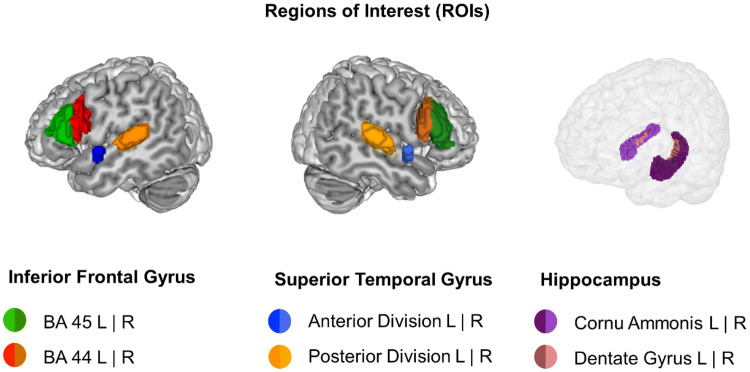
Regions of interest (ROIs). ROIs were defined on the Jülich Histological (IFG and Hippocampus) and Harvard–Oxford (superior temporal gyrus) Atlases. Population map for all areas was truncated at 50%. *BA* Brodmann’s area, *L* left, *R* right

To investigate the role of IFG, hippocampus, and STG in the processing of hierarchical structures, we extracted 8 ROIs from Jülich Histological Atlas comprising IFG (BA 44 and 45, left and right) (Amunts and Zilles 2012), hippocampus (Cornu Ammonis and Dentate Gyrus, left and right) (Amunts et al. 1999) (Fig. 5), and 4 ROISs from the Harvard–Oxford atlas (anterior and posterior STG, left and right) (Fischl et al. 2004). The population map of these regions was truncated at 50%.

Using the model structure of the flexible factorial within-subject ANOVAs described above, we extracted the mean of the single-subject beta values across each ROI mask using the REX toolbox (https://web.mit.edu/swg/software.htm), with global scaling. We then computed linear mixed models with these beta values as dependent variable, with RULE (Recursion, Repetition and Iteration) as within-factor in the generation phase, and RULE, CORRECTNESS (Correct, Foil) and their interaction in the test sound phase. Statistical analyses were performed in R studio (1.1.453). Models were computed with the function lmer() with package lme4 (Bates et al. 2014) using participants as random factor. Models are reported using ANOVA (type = II) and the R package Anova() for *p* values. When main effects were found, we tested for pairwise differences with emmeans() (Russell 2018), using Kenward-Roger methods to calculate the degrees of freedom, and Tukey *p* value adjustment when comparing 3 parameters.

## Results

### Pretesting

Our sample scored on average 69% (SD = 14) in the Melodic Memory Task, a result within the normative range for non-musicians in the United Kingdom (Müllensiefen et al. 2014).

Results for the discrimination 2-forced choice task used to increase experience with the stimulus material showed an average percentage of correct answers of 77% (SD = 20) in the Iteration Rule and 85% (SD = 17) in the Recursion Rule. Participants could correctly reject all foil categories (accuracy > 70% for all), establishing that they were not using simple heuristics to accomplish successful discrimination (see “Methods” for details).

On the same day as the MR testing, participants performed one session of 18 trials with the same 1-forced choice detection task used within the scanner (but with three foil categories instead of one). Mean accuracy was 73% in Iteration trials, 73% in Recursion and 82% in Repetition. The corresponding discriminability values (*d*’) were 0.92 for Iteration (SD = 0.60), 0.83 for Recursion (SD = 0.70), and 1.08 for Repetition trials (SD = 0.36), indicating that participants discriminated well above chance levels.

### Behavioral

During MRI scanning, participants scored on average 75% in Iteration trials (SD = 23), 79.2% in Recursion (SD = 18) and 89% in Repetition (SD = 7). The corresponding discriminability values (*d*’) were 0.88 for Iteration (SD = 0.91), 1.06 for Recursion (SD = 0.79), and 1.31 for Repetition trials (SD = 0.37). We found no significant effects of RULE on discriminability scores (*F*(2, 28) = 2.2. *p* = 0.13, within-subjects ANOVA).

Response time in the decision phase was on average 740 ms in Iteration trials (SD = 290), 780 ms in Recursion (SD = 330) and 580 ms in Repetition (SD = 140). We found an effect of RULE on response time (*F*(2, 28) = 6.2. *p* = 0.006), specifically there was a statistically significant difference between Repetition and both Recursion and Iteration (both *p* values < 0.02) but not between Iteration and Recursion (*p* = 0.5).

### fMRI

#### Generation phase

**Fig. 6:**
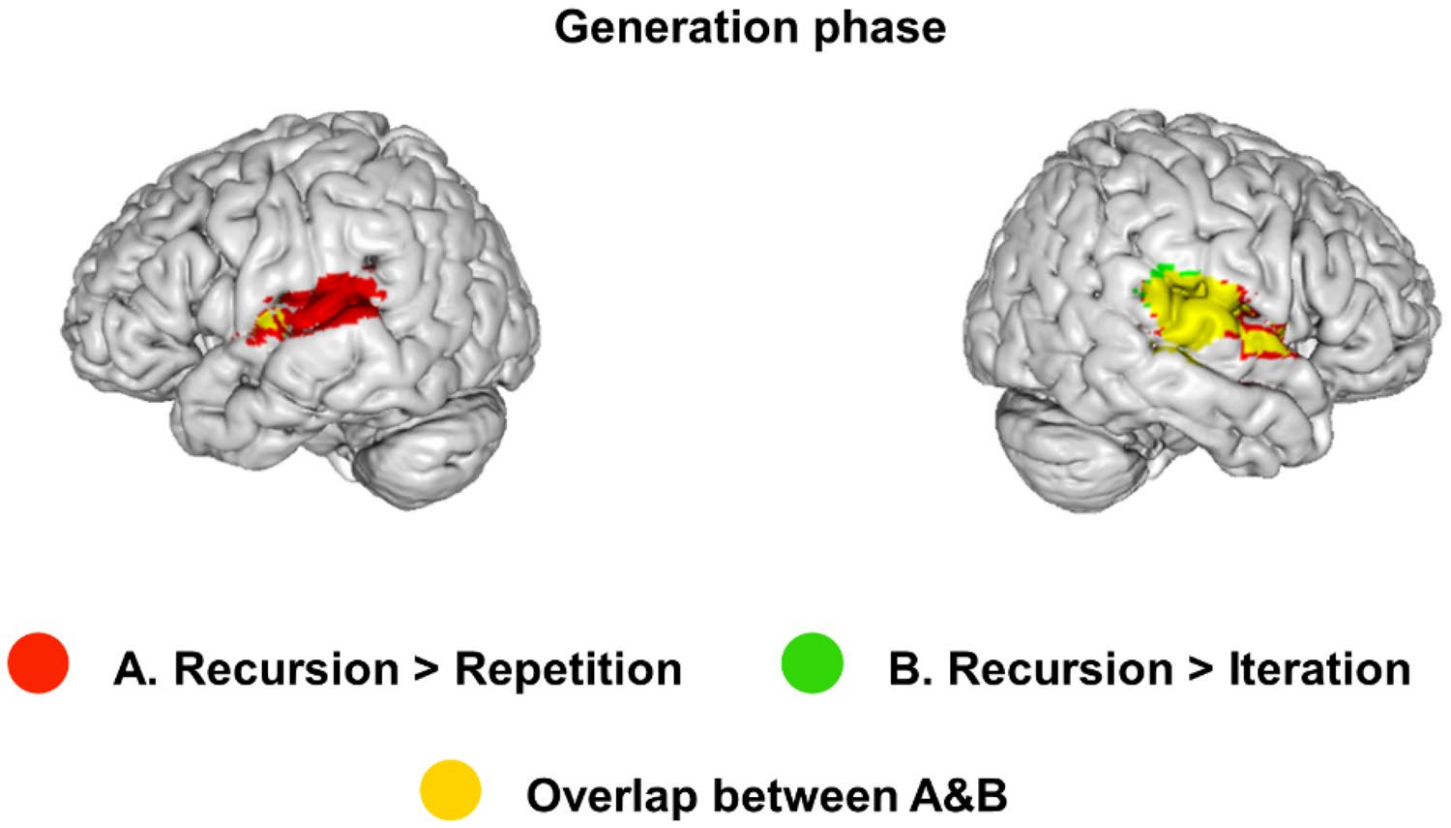
Brain activation during the generation phase (between steps 3 and 4)

Data is depicted in Fig. 6 and Table 1. In comparison with Repetition, we found that imagining new hierarchical levels using the Recursion rule activated a bilateral network comprising Planum Temporale (PT) and Heschl’s Gyrus (HG), extending to the posterior superior temporal gyrus (STG). The linear contrast Recursion > Iteration yielded a similar pattern on the right hemisphere, but did not extend to PT and pSTG on the left. The contrasts Iteration > Repetition and Iteration > Recursion yielded no significant activations.The application of the Recursion rule during the generation phase yielded specific activations in contrast with both the simple Repetition and Iterative rule. Compared to Repetition, the Recursive rule activated a bilateral network comprising Heschl’s Gyrus (HG), posterior Superior Temporal Gyrus (pSTG) and Planum Temporale (PT). The same network was active in the contrast Recursion > Iteration for the right hemisphere, but for the left hemisphere, activity was restricted to the HG.

**Table 1:** Rule effect in the generation phase

Region	Hem	*k*	*x*	*y*	*z*	*T* value
Recursion > Iteration						
pSTG	R	584	70	− 26	10	6.06
			66	− 20	2	5.93
HG			56	− 10	4	4.13
HG	L	243	− 48	− 16	2	4.86
Central opercular cortex			− 50	− 6	12	4.20
Insular cortex			− 38	− 8	12	3.61
Recursion > Repetition						
pSTG	R	1055	66	− 20	4	6.72
PT			56	− 24	6	6.10
HG			52	− 14	4	5.52
PT	L	807	− 48	− 18	4	5.58
pSTG			− 58	− 34	8	5.15
HG			− 56	− 10	2	4.90

Whole-brain activation cluster sizes (*k*). MNI coordinates (*x*. *y*. *z*) and *T* values for the Rule contrast in the execution phase (*p*_voxel_ < 0.001; *p*_cluster_ < 0.05. FWE corrected). Repeated labels within each cluster are not depicted

*Hem* hemisphere, *HG* Heschl’s Gyrus, *pSTG* superior temporal gyrus, posterior division, *PT* planum temporale

**Fig. 7:**
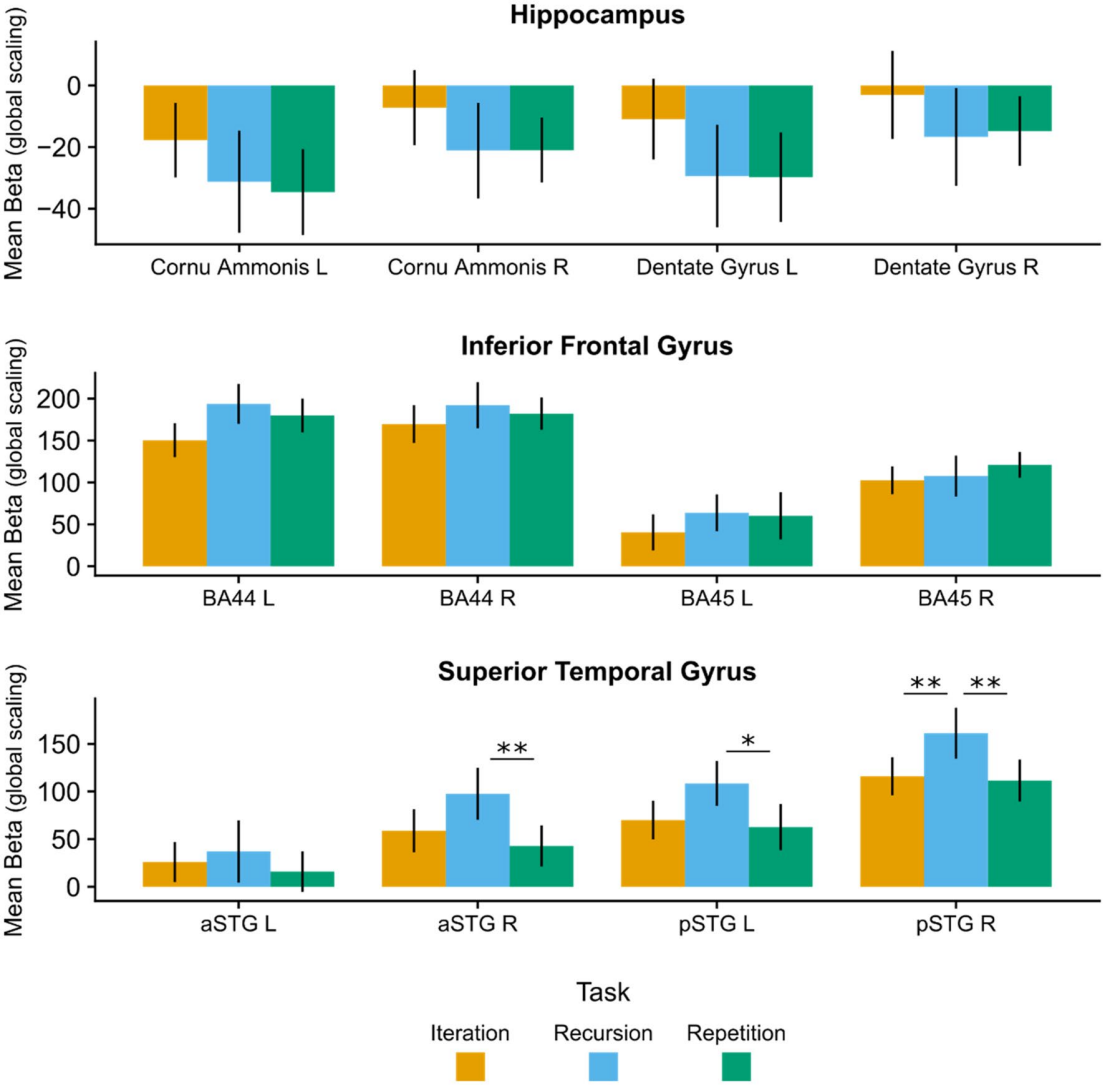
ROI analysis during the generation phase. For each of the 12 ROIs, we performed linear mixed models for single-subject beta values, with Rule as fixed factor. We found significant main effects of Rule only within the STG (see text for details). In particular, Recursion activity was higher than Iteration only within right posterior Superior Temporal Gyrus [*t*(28) = 3.3, *p* < 0.001]. *p* posterior, *a* anterior, *L* left, *R* right, *STG* superior temporal gyrus, *IFG* inferior frontal gyrus. *p < 0.05 **p < 0.001. Mean Beta (global scaling): mean beta values divided by the global mean across all voxels and scaled to 100

To test whether the IFG, STG or hippocampus, or any of their sub-regions played a significant role in the generation of melodic hierarchies, we performed ROI analyses. In particular, we tested whether the mean activation differed between rules for each ROI individually (Fig. 7). Significant main effects of Rule were found only for right anterior STG [aSTG R; *F*(2,28) = 7.7, *p* < 0.001], right posterior STG [pSTG R; *F*(2,28) = 8.1, *p* < 0.001] and left posterior STG [pSTG L: *F*(2,28) = 4.1, *p* = 0.03]. Within all other ROIs the effect of Rule was not significant (all *p*s > 0.05). In particular, we found that mean activity for Recursion was higher than for Repetition in all three regions [aSTG R: *t*(28) = 3.2, *b* = 1.0, *p* < 0.001; pSTG R: *t*(28) = 3.6, *b* = 0.9, *p* < 0.001; pSTG L: *t*(28) = 3.7, *b* = 0.9, *p* = 0.03], and Recursion activity was higher than Iteration within pSTG R [*t*(28) = 3.3, *b* = 0.9, *p* < 0.001].

Comparing ROI mean activity can potentially conceal interesting activity cluster differences between rules within each ROI. To address this issue, we ran several Small Volume Correction (SVC) analyses within the same ROI masks. Other than the results already reported for mean ROI activity there were no other significant differences during the generation phase (with uncorrected *p* < 0.01).

#### Test sound phase

**Fig. 8:**
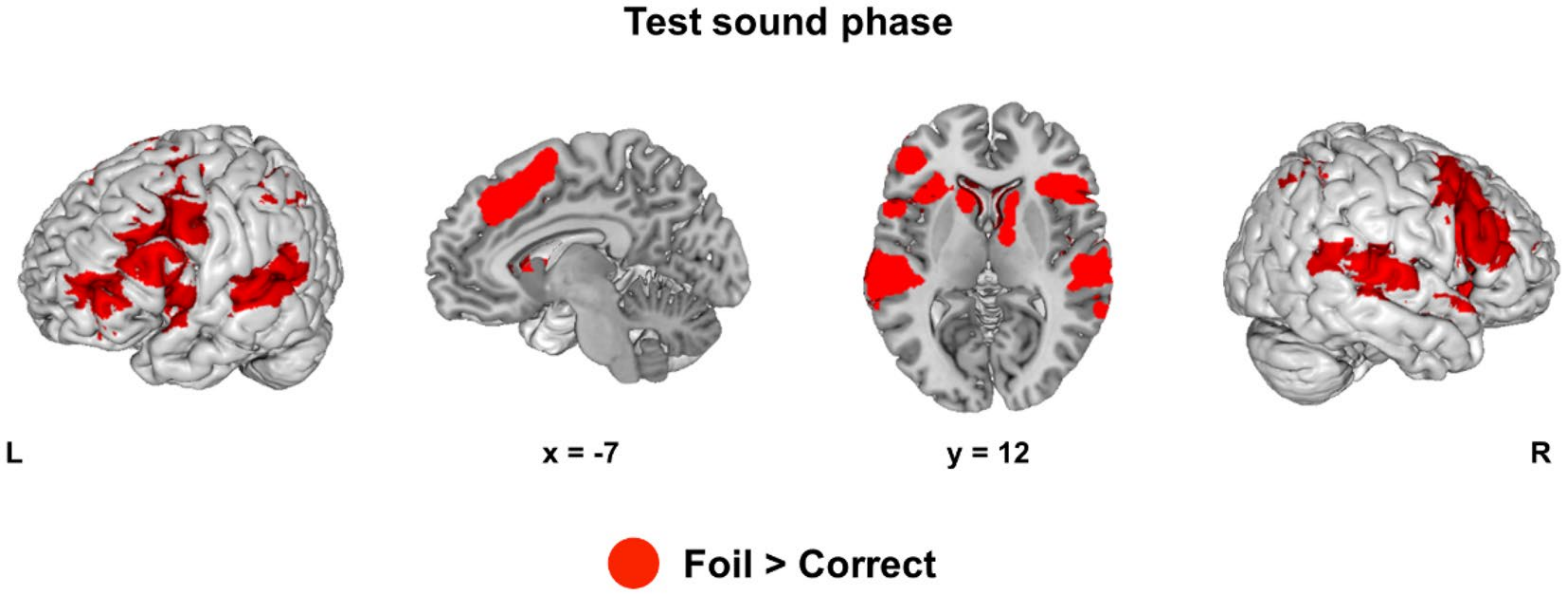
Brain activation during the test sound phase (first part of step 4). A fronto-temporo-parietal network was activated when participants heard sequences of tones that violated the underlying rule (Foil) in comparison with well-formed tone sequences (Correct) (main effect of CORRECTNESS). This finding was consistent across all rules (Iteration, Recursion and Repetition). There was no effect of RULE in the test sound phase and no interaction between RULE and CORRECTNESS

Data is depicted in Fig. 8 and Table 2. In the test sound phase, we modelled both Rule and Correctness (Correct vs. Foil). We found no main effect of Rule and no interaction between Rule and Correctness. In this phase, we found that when participants heard melodic sequences that violated the correct rules (vs. well-formed sequences), there was activity in a bilateral fronto-temporo-parietal network. This network included clusters in the Superior Frontal Gyrus (SFG) extending to Paracingulate Gyrus, Middle Frontal Gyrus (MFG) extending to IFG, STG, Supra Marginal Gyrus (SMG), Angular Gyrus (AG) extending to Lateral Occipital Cortex, and finally a Supplementary Motor Cortex cluster extending to the basal ganglia.

**Table 2:** Rule effect in the test sound phase

Region	Hem	*k*	*x*	*y*	*z*	*T* value
Foil > Correct						
MFG	R	4633	48	12	40	6.98
IFGpo			58	18	18	6.56
			42	14	32	6.53
SFG	L	2247	0	30	44	6.90
Paracingulate cortex			− 6	18	46	6.50
MFG	L	4131	− 38	12	28	6.67
			− 38	0	52	5.78
Frontal pole			− 46	40	8	5.57
pSTG	L	1135	− 64	− 26	6	6.20
PT			− 56	− 30	6	5.68
pSMG			− 62	− 42	12	5.29
pSMG	L	999	− 42	− 46	42	5.90
			− 32	− 56	36	5.70
pSMG	L	514	36	− 46	36	4.73
AG			44	− 50	46	4.39
Lat. occipital			40	− 58	46	4.27
Supplementary motor	R	234	12	− 2	4	4.27
Caudate			12	16	2	3.79
Pallidum			12	6	0	3.69

Whole-brain activation cluster sizes (*k*). MNI coordinates (*x*. *y*. *z*) and *Z* scores for the Rule contrast in the execution phase (*p*_voxel_ < 0.001; *p*_cluster_ < 0.05. FWE corrected). Repeated labels within each cluster are not depicted

*Hem* hemisphere, *Lat* Lateral, *pSTG* Superior temporal Gyrus, posterior division, *SFG* superior frontal gyrus, *MFG* middle frontal gyrus, *IFG po* inferior frontal gyrus, pars opercularis, *PT* planum temporale, *AG* angular gyrus, *pSMG* supramarginal gyrus, posterior division

**Fig. 9:**
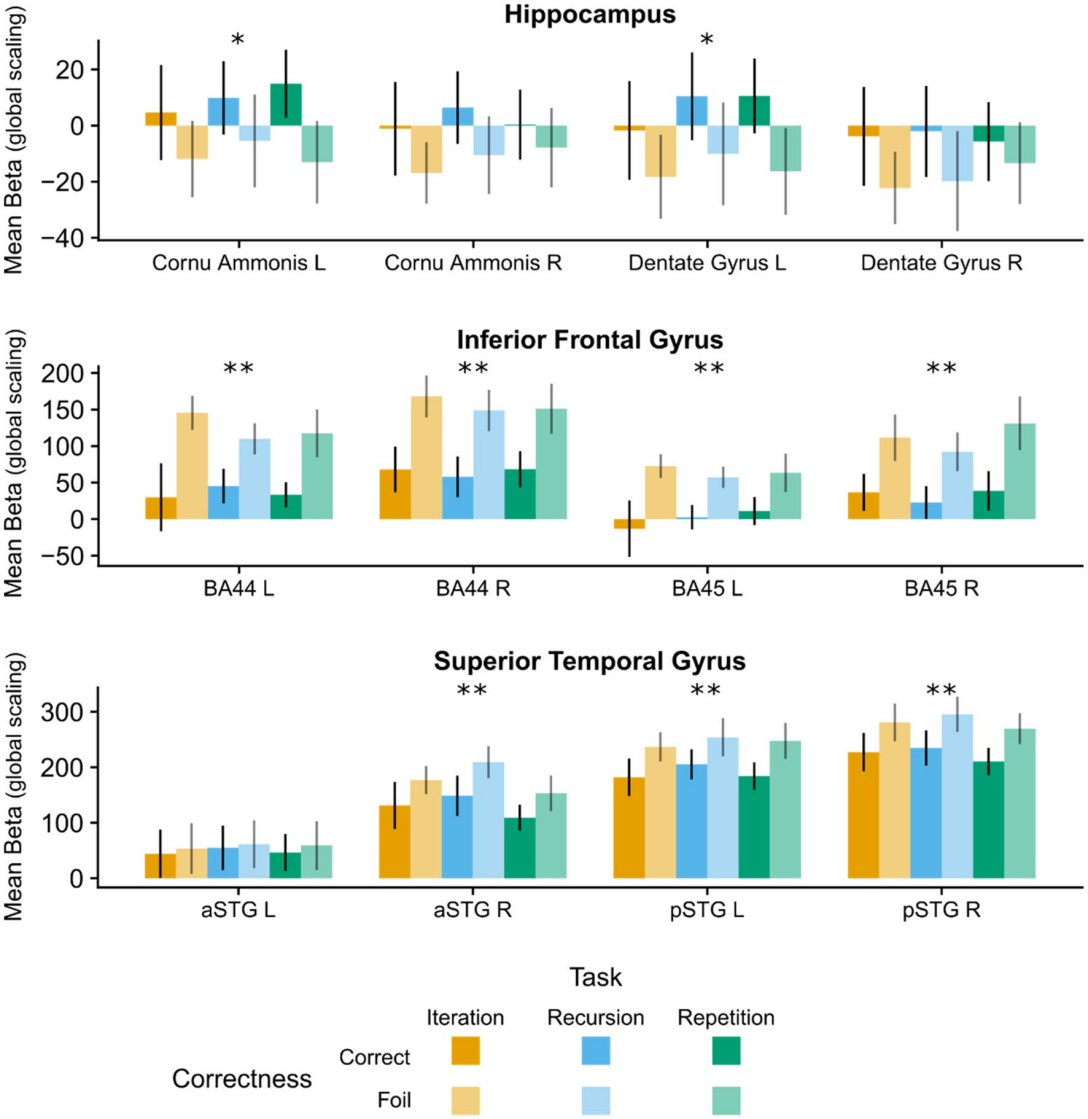
ROI analysis during the test sound phase. Mimicking the whole brain analysis, we found an increase of activity in the contrast Foil > Correct in all IFG sub-regions, right anterior and posterior STG, and left posterior STG (all *p* < 0.001). In addition, the same contrast was associated with a decrease in activity in the left hippocampal subregions Cornu Ammonis and Dentate Gyrus (both *p* = 0.02). Finally, we found a main effect of Rule within the right anterior STG, in particular, activity was higher in Recursion than Repetition [*t*(70) = 2.6, *p* = 0.04]. *p* posterior, *a* anterior, *L* left, *R* right, *STG* superior temporal gyrus, *IFG* inferior frontal gyrus. *p < 0.05 **p < 0.001. *Mean Beta (global scaling)* mean beta values divided by the global mean across all voxels and scaled to 100

In addition to whole brain analysis, we performed ROI analyses, using the same regions as for the generation phase (Fig. 9). For each region, we performed a linear mixed model with Rule, Correctness and their interaction as fixed factors, and participant as random factor. Within the left hippocampus, we found a main effect of Correctness in the Cornu Ammonis [*F*(1,70) = 5.7, *p* = 0.02] and [Dentate Gyrus *F*(1,70) = 5.4, *p* = 0.02]. In particular, activity in these regions was *higher* during processing of Correct tone sequences vs. Foils [Cornu Ammonis: *t*(70) = 2.4, *b* = 0.5, *p* = 0.02; Dentate Gyrus: *t*(70) = 2.3, *b* = 0.9, *p* = 0.02]. Similarly, with the exception of left anterior STG, we found a main effect of Correctness within all IFG regions and STG regions, [BA44 L: *F*(1,70) = 27.5, *p* < 0.001; BA44 R: *F*(1,70) = 42.9, *p* < 0.001; BA45 L: *F*(1,70) = 19.1, *p* < 0.001; BA45 R: *F*(1,70) = 34.2, *p* < 0.001; aSTG R: *F*(1,70) = 11.1, *p* < 0.001; pSTG L: *F*(1,70) = 25.3, *p* < 0.001; pASTG R: *F*(1,70) = 35.8, *p* < 0.001]. Contrary to the hippocampus, activity in these regions was lower during the processing of Correct tone sequences vs. Foils, [BA44 L: *t*(70) = − 5.2, *b* = − 2.2, *p* < 0.001; BA44 R: *t*(70) = − 6.6, *b* = -2.2, *p* < 0.001; BA45 L: *t*(70) = − 4.4, *b* = − 1.6, *p* < 0.001; BA45 R: *t*(70) = − 5.9, *b* = -1.9, *p* < 0.001; aSTG R: *t*(70) = − 3.3, *b* = − 1.2, *p* = 0.001; pSTG L: *t*(70) = − 5.0, *b* = − 1.4, *p* < 0.001; pSTG R: *t*(70) = − 6.0, *b* = − 2.2, *b* = − 1.4, *p* < 0.001].

Finally, we found a main effect of Rule within the right anterior STG [*F*(2,70) = 3.4, *p* = 0.04]. In particular, activity was higher in Recursion than Repetition [*t*(70) = 2.6, *b* = 1.2, *p* = 0.04]. We found no other significant main effects or interactions within the ROIs.

As in the generation phase, we performed SVC analyses to detect whether there were particular clusters of activity differentiating task rules in addition to the mean ROI analysis. We replicated the mean ROI results and found additional activity clusters within the left posterior STG for the contrast Recursion > Iteration (*T* = 3.58, *x* = − 66, *y* = − 22, *z* = 6, *K* = 8, *p*_FWE-cluster_ = 0.028), and within the right posterior STG for the contrast Recursion > Repetition (*T* = 3.54, *x* = 66, *y* = − 14, *z* = 0, *K* = 6, *p*_FWE-cluster_ = 0.031). There were no other significant clusters within STG, hippocampus or IFG (with threshold uncorrected *p* > 0.01).

## Discussion

Our goal was to isolate and investigate the neural bases supporting the generation and representation of hierarchies in melodic sequences. To that aim, we devised a paradigm in which identical melodic sequences were generated according to different rules: A Recursion rule, which added hierarchical levels via recursive embedding; an Iterative rule, which successively added items to a fixed hierarchical level, without creating new levels; and a control Repetition rule, which simply required short term memory of a complete melodic sequence without any cognitive transformation. In our procedure, we primed participants with a certain rule by successively presenting three melodic sequences corresponding to the first three steps that resulted from the application of each rule. After the third step, we asked participants to apply the rule one step further and to imagine the next melodic sequence (generation phase). Then we presented a fourth sequence (test sound phase), correct or foil, and asked participants to evaluate whether it matched their predictions. Using this paradigm we could isolate the neural structures active in the representation of Recursive Hierarchical Embedding (RHE), both in anticipation (generation phase) and during the perception (test sound phase) of a melodic hierarchy.

As in our previous behavioral work (Martins et al. 2017), we found that, after training, participants were able to achieve comparable accuracy in the Recursion and Iteration rules, and to reject different foil categories as incorrect, indicating that they did not rely upon any simple auditory heuristic to solve the tasks. They rejected different foil categories in both a 2-forced choice discrimination task and a 1-choice detection task, similar to the fMRI procedure. Crucially, the Recursion rule in the auditory paradigm has been shown behaviorally to share cognitive resources with similar tasks in the visual and action sequencing domains (Martins et al. 2017). This suggests that the Recursion rule in our auditory task is adequate to isolate the mechanisms underlying the representation of RHE. In the following paragraphs, we summarize the results we obtained using this reliable methodology.

First, we found that during the generation phase, between steps 3 and 4, there was increased activity in STG, HG and PT for the Recursion rule, in comparison with Iteration and with Repetition, indicating that these regions are particularly involved in the cognitive generation of new hierarchical levels. This activity was more robust in the right hemisphere. Based on previous hypotheses implicating IFG, STG and hippocampus in the processing of hierarchies across domains (see introduction for a review), we specifically tested whether these regions were active in the generation phase. In line with whole brain analysis, we found significantly increased activity only in the right posterior STG for both contrasts Recursion vs. Iteration and Recursion vs. Repetition.

Second, during the test sound phase, during which participants listened to melodic sequences that were identical across conditions, the whole brain analysis revealed no significant difference between task rules. However, ROI analyses suggest that some clusters within the posterior STG are more active in Recursion than in either Iteration (left hemisphere) or in Repetition (right hemisphere), thus partially replicating our results for the generation phase.

In addition, we found strong effects of Correctness, meaning that participants evaluated whether their expectations were met. In line with previous studies (Koelsch et al. 2005; Musso et al. 2015; Salimpoor et al. 2015; Seger et al. 2013), in this analysis we found increased activity in a fronto-temporo-parietal network during the processing of violations relative to well-formed structures. Our ROI analyses confirmed this finding for each subregion within IFG (BA 44 and 45, both left and right) and STG (pSTG L, aSTG R, pSTG R). Interestingly, processing violations also decreased activity across several regions within left hippocampus.

We now turn to a discussion of how these results contribute to the understanding of the neural bases of the representation of recursive hierarchical embedding rules.

### STG in the generation of new levels in melodic hierarchies

Pervious research has implicated both IFG and STG in the processing of music syntax (Koelsch et al. 2005, 2002; Minati et al. 2008; Musso et al. 2015; Seger et al. 2013). However, the specific roles of these areas in the generation of hierarchies remain unknown (Bianco et al. 2016; Fadiga et al. 2009; Fitch and Martins 2014; Friederici 2011; Koelsch et al. 2002; Maess et al. 2001; Makuuchi et al. 2009; Patel 2003; Zaccarella et al. 2015).

In our experiment, STG (especially the right posterior STG) was robustly more active in both Recursion > Iteration and Recursion > Repetition during the stimulus-free generation phase. However, activity in this area did not differ between Iteration and Repetition, suggesting that this effect is more likely to reflect hierarchical generative effort than any simple difference in in the number of tones maintained in memory. This region is not only associated with the processing of music syntax, but it is more generally thought to be a repository of tonal sequence schemas (Janata 2002) and tonal relations, being active in auditory imagery and prediction tasks (Salimpoor et al. 2015, for a review). Relevant for our task, STG has also been shown to differentiate between ascending and descending melodic contours (Lee et al. 2011) and between local and global level violations (Stewart et al. 2008). During the generation phase, participants were required to take step 3, which provides a global context, and to add a new local hierarchical level according to a rule which determined whether this local contour was ascending or descending. Then they were asked to build a guiding prediction of this new structure before the test sound (step 4). Combined with results from the previous literature, our results suggest that the representation of RHE is crucially dependent on the retrieval and manipulation of the appropriate tonal sequence schemas from STG.

In our study, we found no indication that IFG was active in the generation of new hierarchical levels. IFG is commonly active when well-formed structures are contrasted with violations, not only in music syntax (Bianco et al. 2016; Koelsch et al. 2002; Maess et al. 2001; Patel 2003), but also in language (Friederici 2011, for a review). These findings led to the hypothesis that IFG supports the generation of hierarchies across domains (Fadiga et al. 2009; Fitch and Martins 2014; Patel 2003; Fitch 2014). However, instead of supporting functions specific to the generation of hierarchies, IFG might rather support domain-general cognitive functions associated with working memory, cognitive control, or other computations necessary to process unexpected or complex sequences (Bigand et al. 2014; Patel and Morgan 2017; Rogalsky et al. 2011). While our study does not demonstrate a domain-general role of IFG, it is more consistent with this hypothesis than with the hypothesis of a specific role for IFG in the generation of hierarchies.

Finally, against our prior hypothesis, we also did not find hippocampus to be active in the representation of RHE during the generation phase. While this region may be important for the initial formation of new hierarchical schemas (Berens and Bird 2017), with training on a particular category of stimuli, as in the current study, these functions may migrate to other cortical regions, e.g. the superior temporal cortex (Gilboa and Marlatte 2017).

### IFG, STG and hippocampus in the processing of melodic sequences

In contrast to the generation phase, we did not find significant differences in brain activity between Recursion and Iteration when participants heard the melodic sequences during the test sound phase. Because brain activity in all trial phases (i.e. generation, test sound, and decision) was included in the first level GLM, this means that this phase did not explain additional rule differences when the generation phase was accounted for. In other words, the stimuli themselves were not represented differently once the effects of expectancy were also modelled.

Although we did not find an effect of rule, we replicated the classical activity pattern for the processing of violations vs. well-formed tonal structures (Koelsch et al. 2005, 2002; Musso et al. 2015; Seger et al. 2013) which included IFG and STG, but also portions of the fronto-parietal network. Again, this pattern of activity seems less likely to reflect increased structural load specific to hierarchical processing, than the recruitment of other mechanisms required to detect and resolve expectancy violations, such as increased attention, cognitive control and auditory working memory (Bigand et al. 2014; Patel and Morgan 2017; Rogalsky et al. 2011).

Another interesting finding in the test sound phase was the deactivation of the hippocampus in the processing of violations. The hippocampus is known to guide reactivation of memory schemas during perceptual experience (Schlichting and Preston 2015), biasing processing from the input system (Gilboa and Marlatte 2017). When new stimuli are presented, these are either assimilated into existing schemas, or the old schemas are modified to accommodate the new stimuli. However, when a certain item strongly violates expectations, activity in the hippocampus is reduced to facilitate violation detection (Armelin et al. 2017). This process might be essential to inhibit accommodation of schemas in response to incorrect stimuli. Interestingly, the accuracy of music schema retrieval is associated with decreased activity in left IFG and increased activity in right hippocampus (Watanabe et al. 2008). Our pattern of activity was symmetrically opposite, hinting that these regions may play complementary roles in detecting and resolving violations.

## Limitations

First, a minor oversight is that we did not balance button press (LEFT, RIGHT) across participants. So there is a cognitive mapping (CORRECT/INCORRECT) to these buttons. We minimized the influence of this design shortcoming by including activity only from the first melodic structure cluster of the test sound phase (we excluded the later part in which the dominant cognitive process was the preparation for response) and by including the button press phase in first level model.

Second, in the fMRI experiment the detection task in the test sound phase was very simple, since there was only one kind of foil: participants only needed to detect ascending vs descending contour. Tasks were simpler in the scanner to keep the stimuli exactly the same across rules: as shown in Fig. 4, a repetition foil in the Recursion condition sounds different than in the Iteration condition. Thus, using these foils in the scanner task would have introduced a perceptual confound. However, participants were trained twice—using both discrimination and detection tasks—with a more complex set of stimuli and with more foils. Thus, it is unlikely that they acquired simple ascending/descending response heuristics, since these would be insufficient to solve the training tasks where they also scored adequately (and equivalently to their performance in the scanner).

Third, accepting that participants were not employing simple heuristics to determine the contour of the fourth level, how can we determine if they were truly able to represent hierarchical relations? Determining the exact computations underlying a behavioral task is always a challenge. However, we surmise that there are minimal representational requirements necessary to solve our task: in the Recursive rule (but not the other rules) participants necessarily have to bind information from two different levels of information: In particular, they have to apply the information derived from a given hierarchical level (with a particular rhythmic structure, pitch range, and melodic contour) to form an expectation about the next level (with a similar but more rapid rhythmic structure, higher pitch range, and same contour). The hierarchical structure is not in the stimulus itself, but rather in the rule that binds each parent tone from level *n* to a set of three children tones in level *n* + 1: the frequency and duration of a parent tone determines the melodic and rhythmic structure of the triplet. Since this relation is a rooted directed acyclical graph with a branched structure, it is by definition hierarchical (Udden et al. 2019). In addition, the previous finding that the ability to perform the Recursive rule strongly and specifically correlates with similar abilities in the visual and motor domains (Martins et al. 2017), supports the assumption that the Recursive task isolates some aspects of hierarchical generativity.

Finally, it could be argued that activity in the generation phase reflects “spillover” activation from steps 1, 2 and 3. However, 1) we modelled all trial phases in first level of analysis, which reduces this spillover and 2) stimuli in these early steps were actually more complex (more items and hierarchical levels) in Iteration and Repetition than in the Recursion rule. Hence, activity in the generation phase is more likely to reflect the additional cognitive load required for the transformation of step 3 into step 4, i.e., the generation of a new hierarchical level, than any long-lasting effects of previous phases. Moreover, patterns of activity in these early trial phases (steps 1, 2 and 3, see Supplementary Materials) clearly show increased activity for Repetition within the Fronto–Parietal Network, and increased activity for both Iteration and Recursion (vs. Repetition) within midline structures. Crucially, in this phase there was no increased activity in Recursion vs. Iteration within the temporal cortices, making it unlikely that spillover effects account for the STG activations we found.

## Conclusion

In this study we used a novel paradigm using musical stimuli to clarify the neural basis of hierarchical cognition in the auditory domain. Previous research has uncovered a peri-sylvian network, incoporating both temporal and frontal cortices, and strikingly similar to that used in language, apparently involved in the processing of musical syntax (Koelsch et al. 2002; Musso et al. 2015; Patel 2003; Sammler et al. 2013). However, these previous studies used a violation paradigm which left unclear whether the observed activations might reflect surprise, attentional change, and cognitive control, rather than factors specific to hierarchical processing of melodic stimuli. Our new paradigm allowed us to tease these factors apart, and revealed robust activation of superior temporal gyrus (particularly right posterior STG), specifically during the process of cognitively generating a new level in an auditory hierarchical structure.

In contrast, inferior frontal regions were only robustly activated when detecting violations versus correct stimuli in all conditions (not specifically for hierarchical generation). This result suggests that the IFG is mostly involved in violation detection/cognitive control in our task, rather than hierarchy generation per se.

This division of labor mirrors recent findings in the visual domain with stroke patients (Martins et al. 2019) and suggests that future work aiming to probe the role of the IFG and STG in music (or other cognitive domains) should use test paradigms that do not rely solely on a violation/correct discrimination, but rather isolate the generative acts involved in processing and manipulating hierarchical representations (Fitch 2014; Fitch and Martins 2014).

From a music cognition perspective, we note that despite their simplicity and limited aesthetic appeal, but thanks to their vertical harmonic structure, our melodic fractals pave the way for the study of musical hierarchies in more complex and musically relevant stimuli, such as musical excerpts making use of contrapuntal techniques.

## Electronic supplementary material

Below is the link to the electronic supplementary material.

Supplementary file1 (PPTX 2810 kb)

Supplementary file2 (DOCX 6717 kb)
