# Supplementary material for: Recursive music elucidates neural mechanisms supporting the generation and detection of melodic hierarchies
Source: Brain Struct Funct. 2020 Jun 26;225(7):1997–2015. doi: 10.1007/s00429-020-02105-7 (PMC7473971; doi:10.1007/s00429-020-02105-7)
Supplement: Supplementary file 1 — Supplementary file1 (PPTX 2810 kb) [file 429_2020_2105_MOESM1_ESM.pptx]

## Slide 1
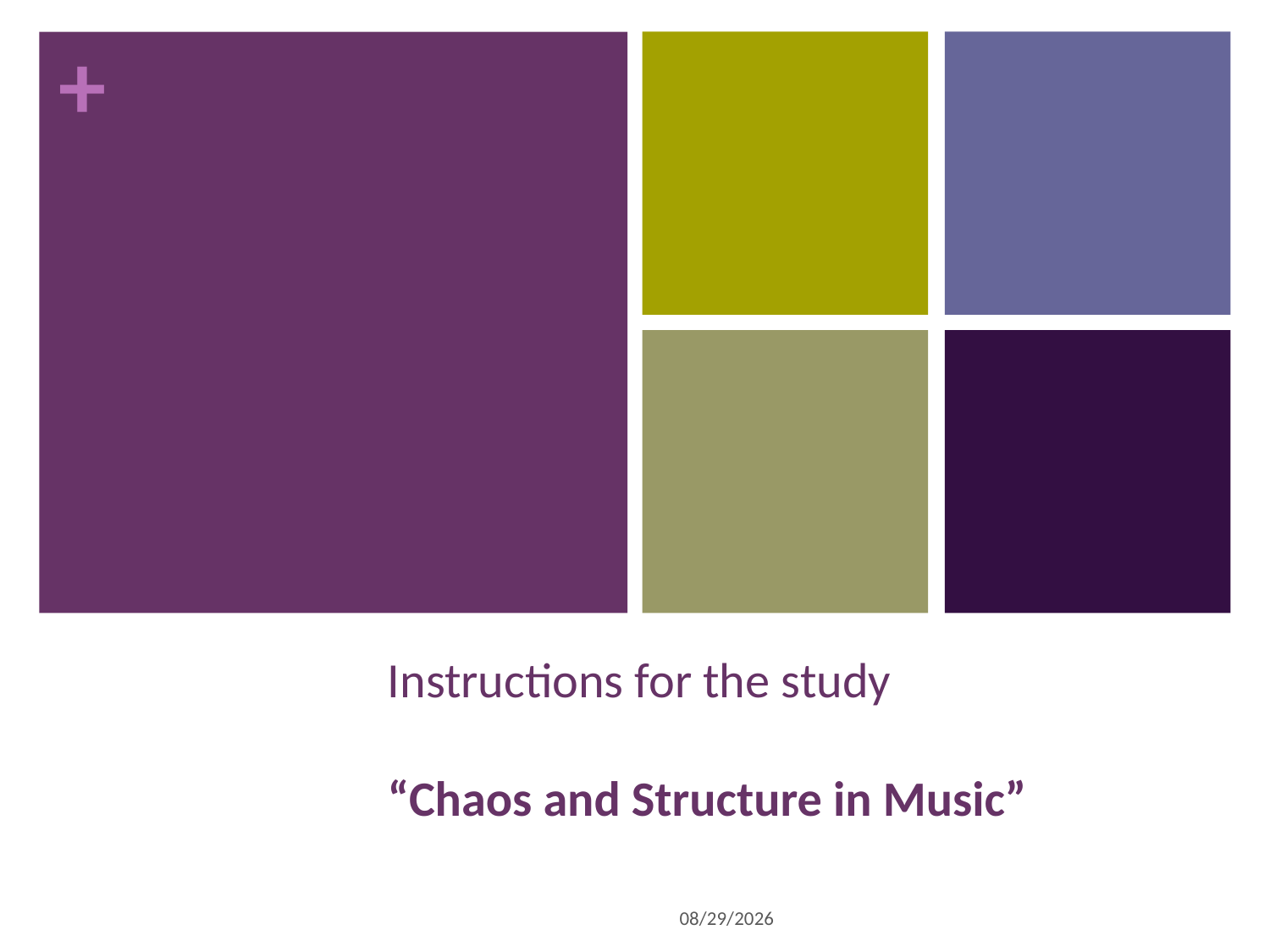

# Instructions for the study “Chaos and Structure in Music”
11/25/13

## Slide 2
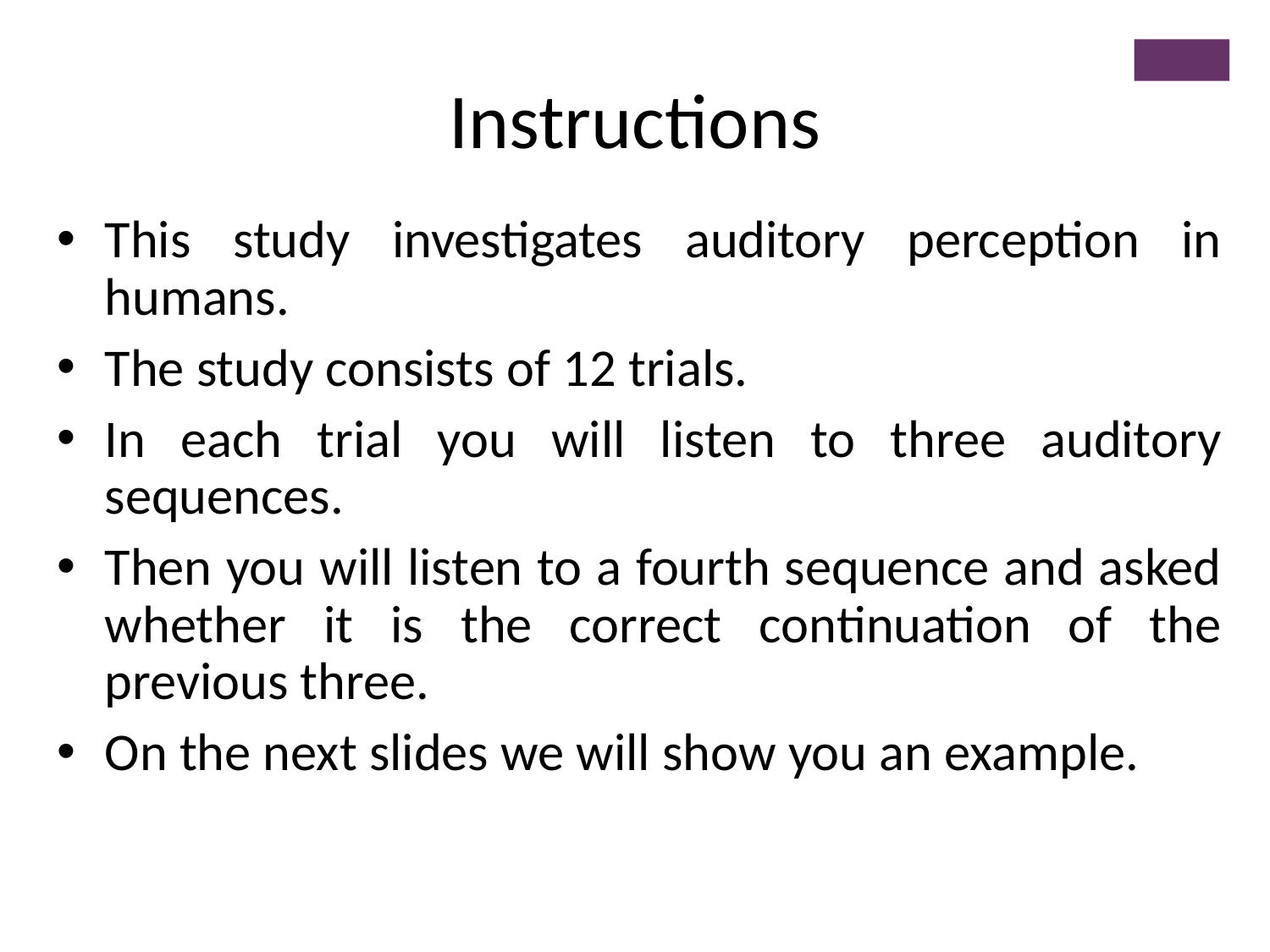

Instructions
This study investigates auditory perception in humans.
The study consists of 12 trials.
In each trial you will listen to three auditory sequences.
Then you will listen to a fourth sequence and asked whether it is the correct continuation of the previous three.
On the next slides we will show you an example.

## Slide 3
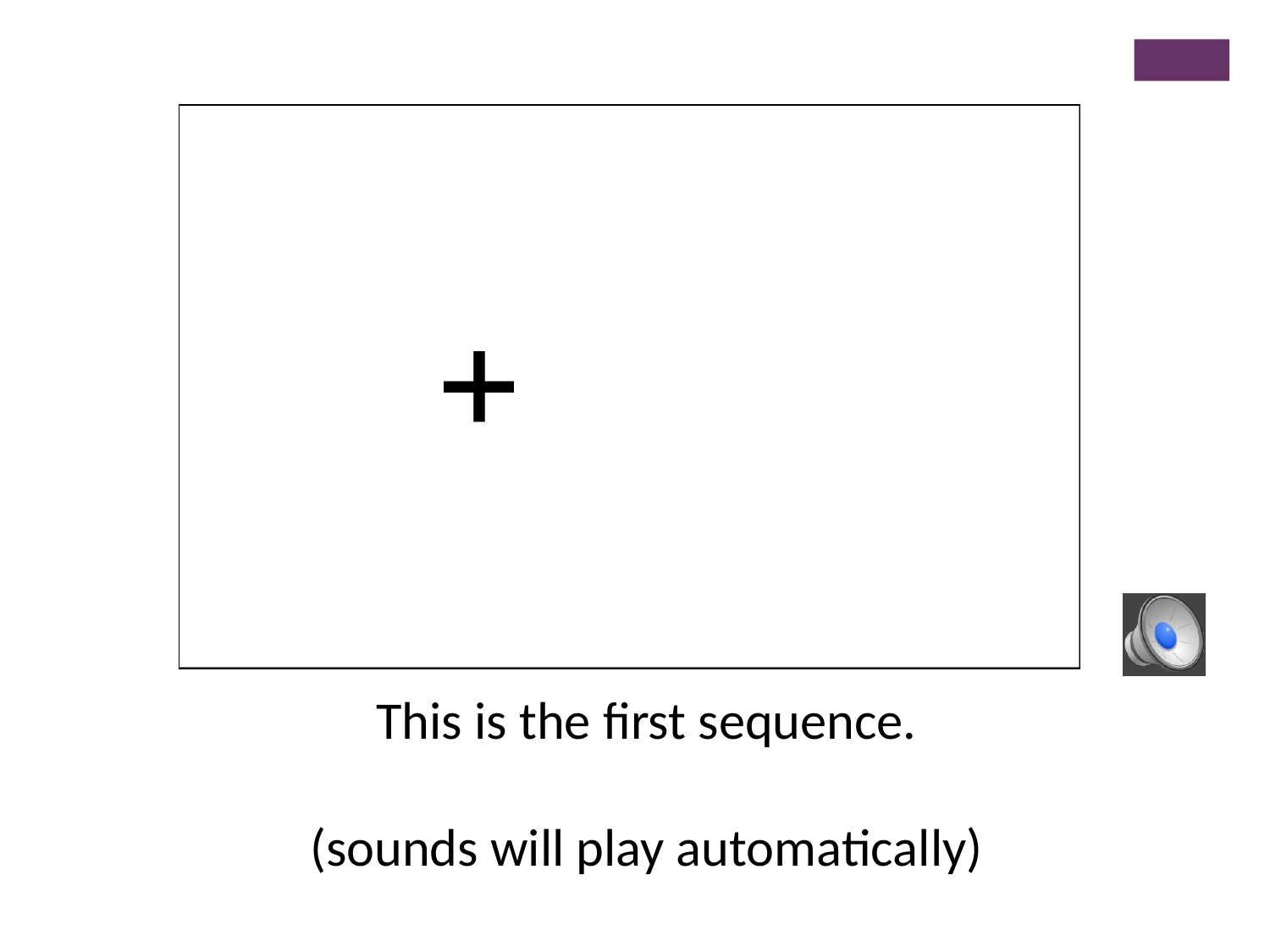

This is the first sequence.
(sounds will play automatically)

## Slide 4
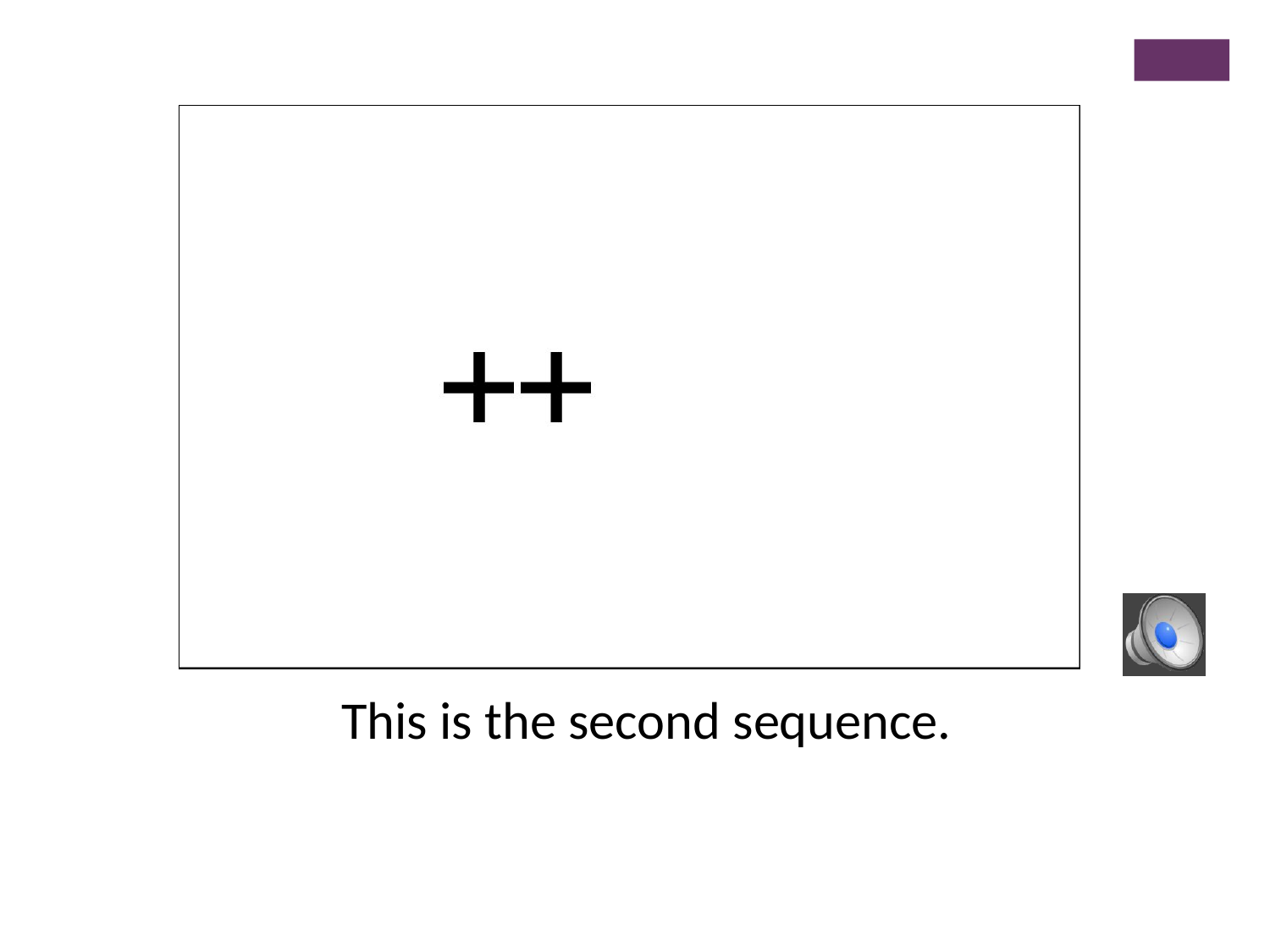

This is the second sequence.

## Slide 5
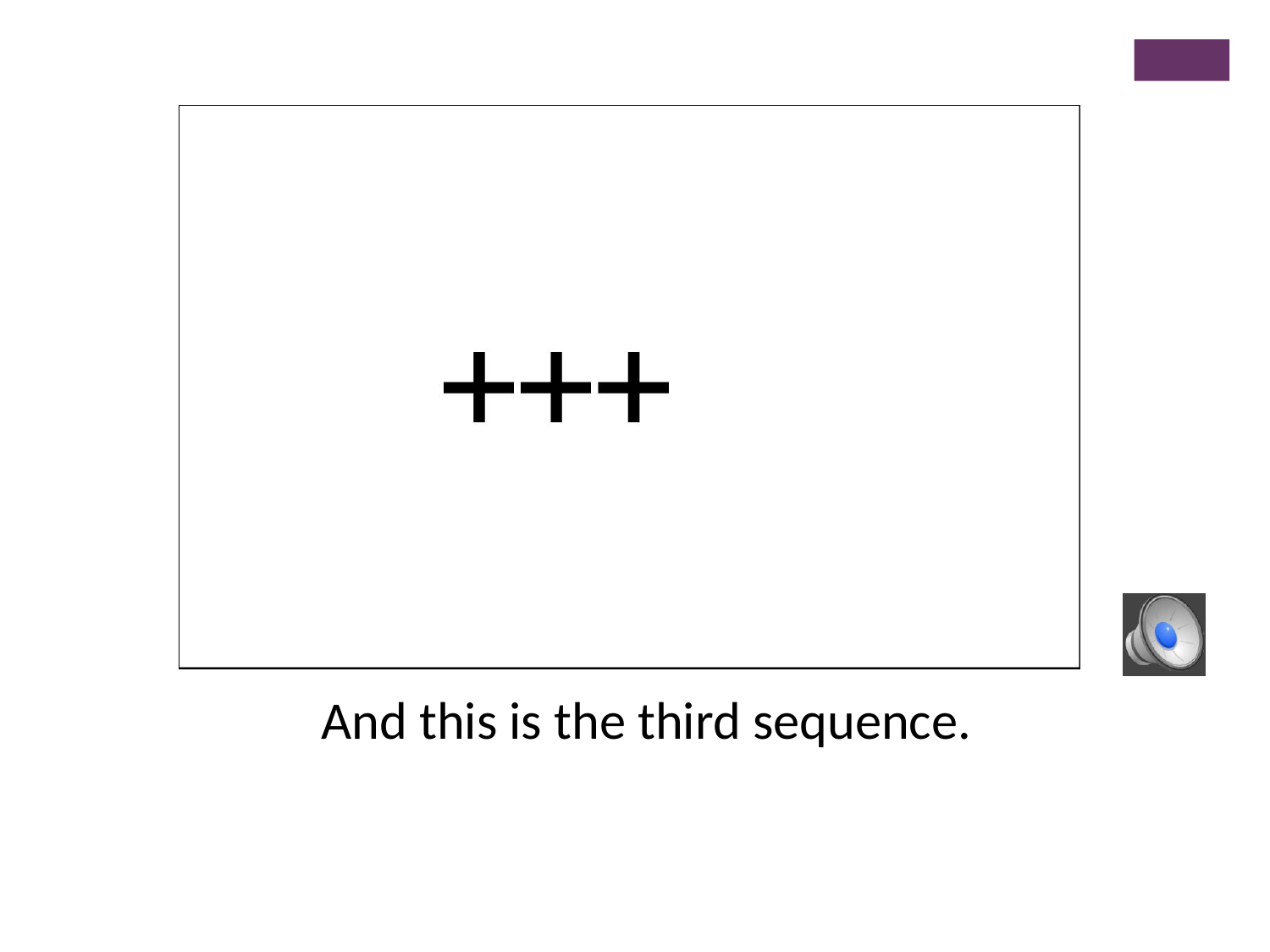

And this is the third sequence.

## Slide 6
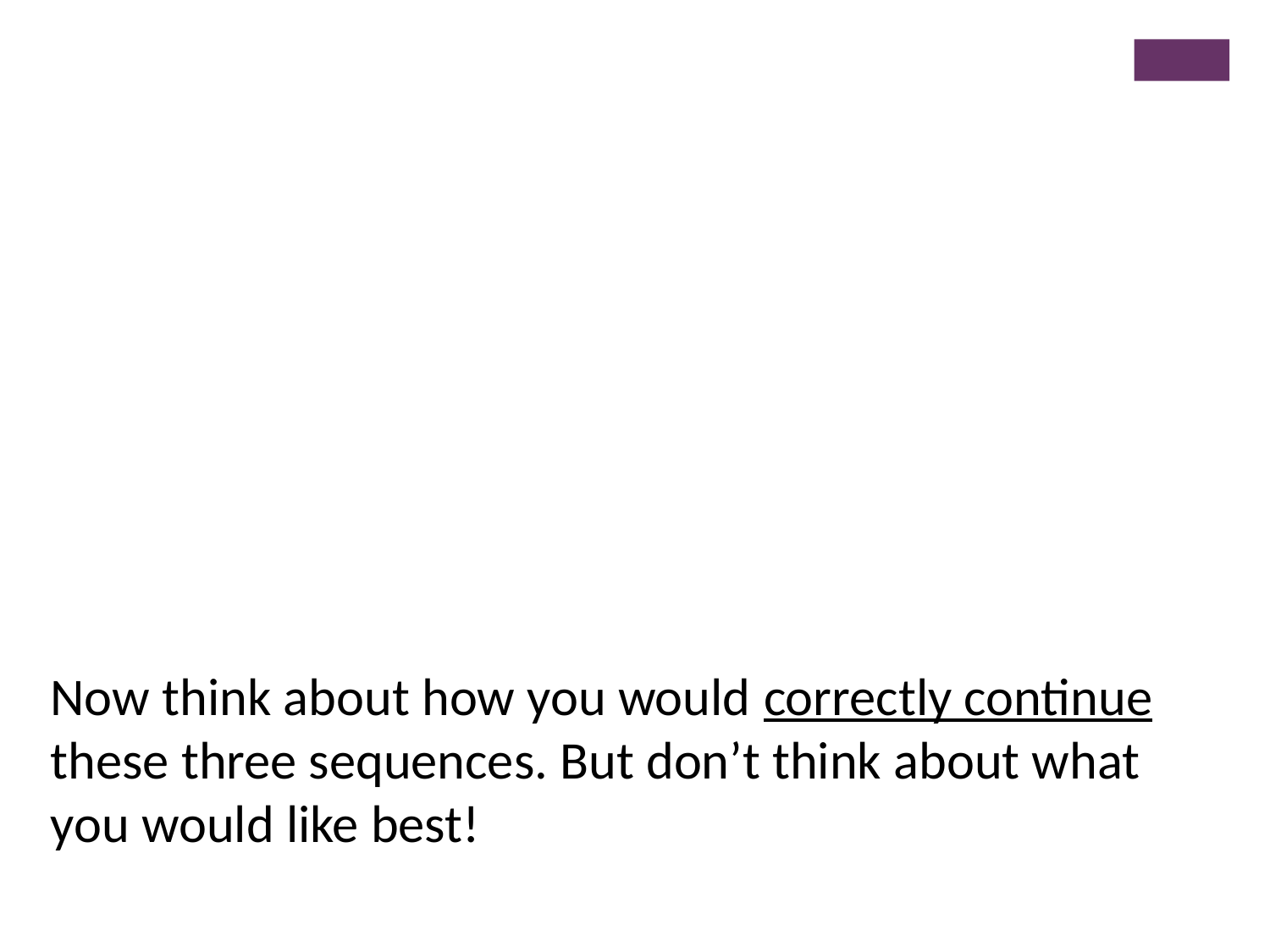

Now think about how you would correctly continue these three sequences. But don’t think about what you would like best!

## Slide 7
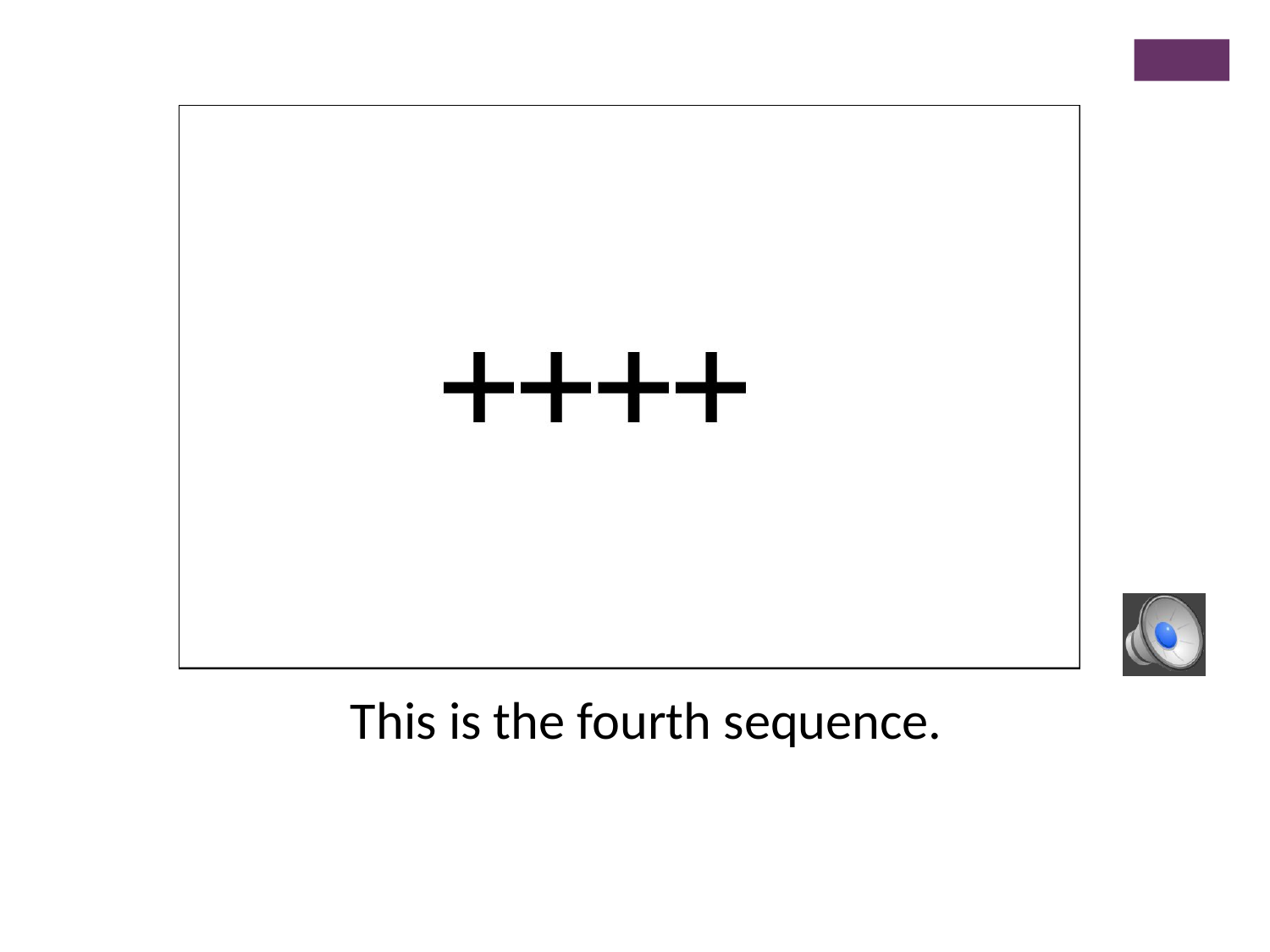

This is the fourth sequence.

## Slide 8
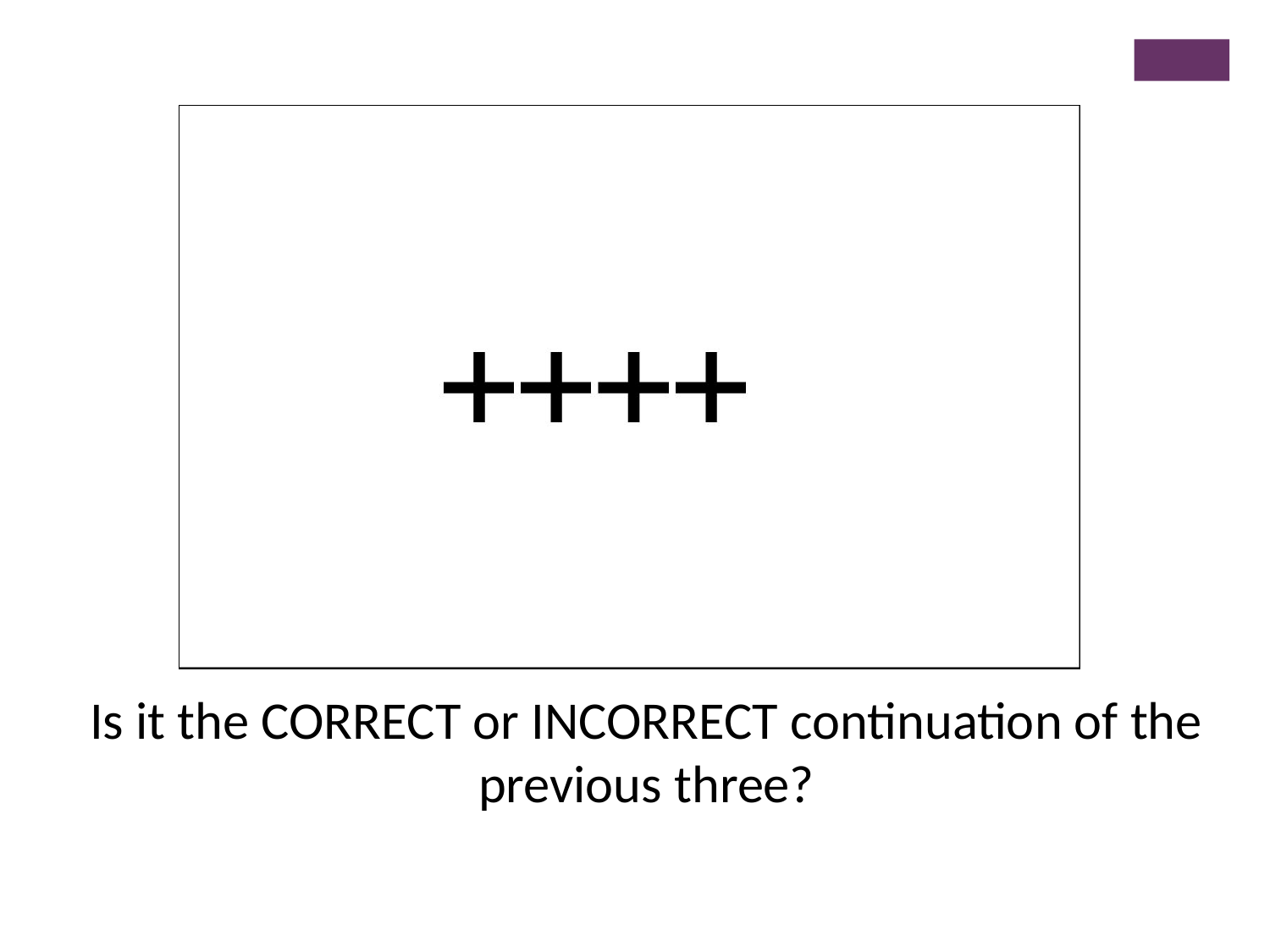

Is it the CORRECT or INCORRECT continuation of the previous three?

## Slide 9
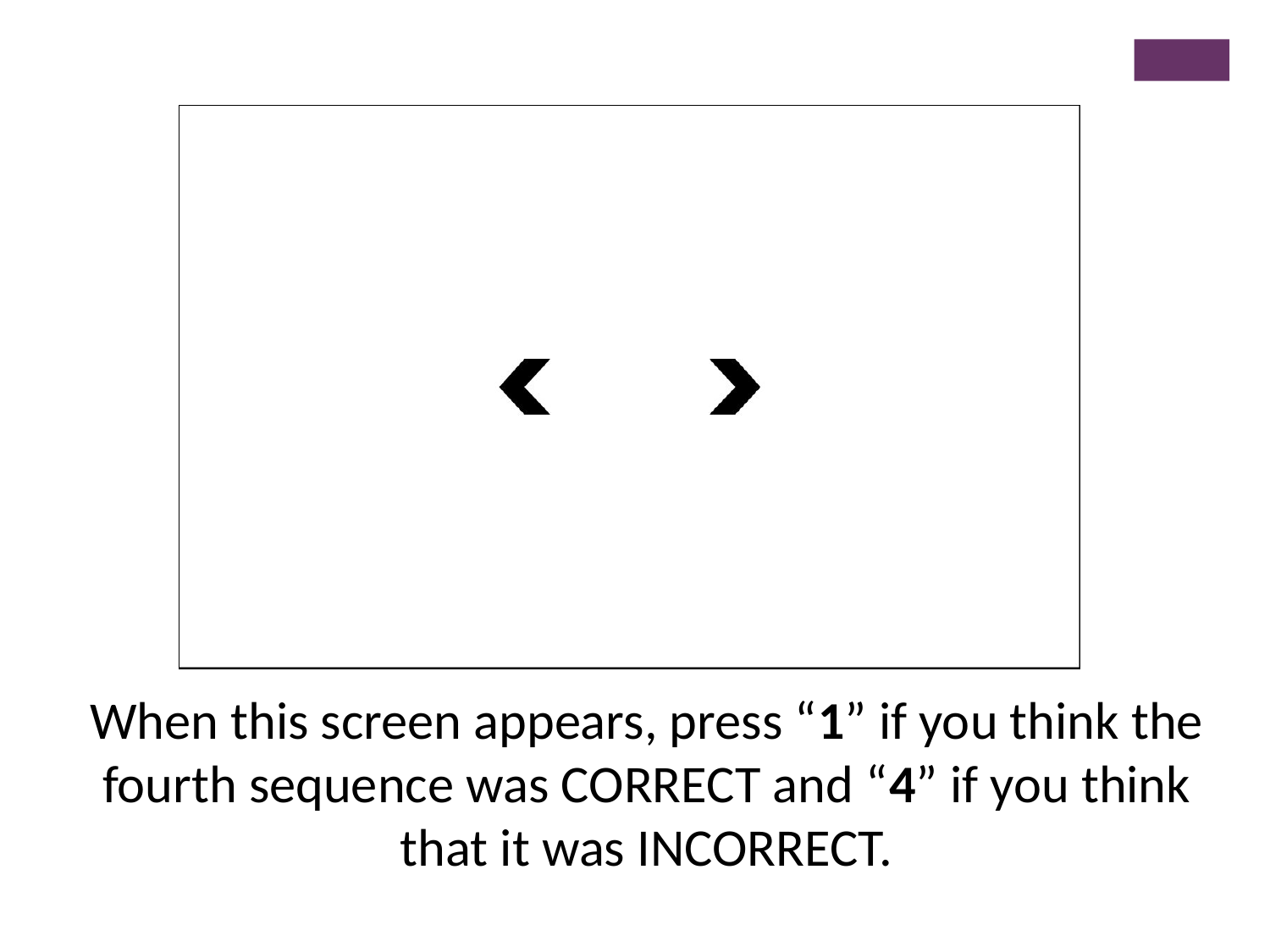

When this screen appears, press “1” if you think the fourth sequence was CORRECT and “4” if you think that it was INCORRECT.

## Slide 10
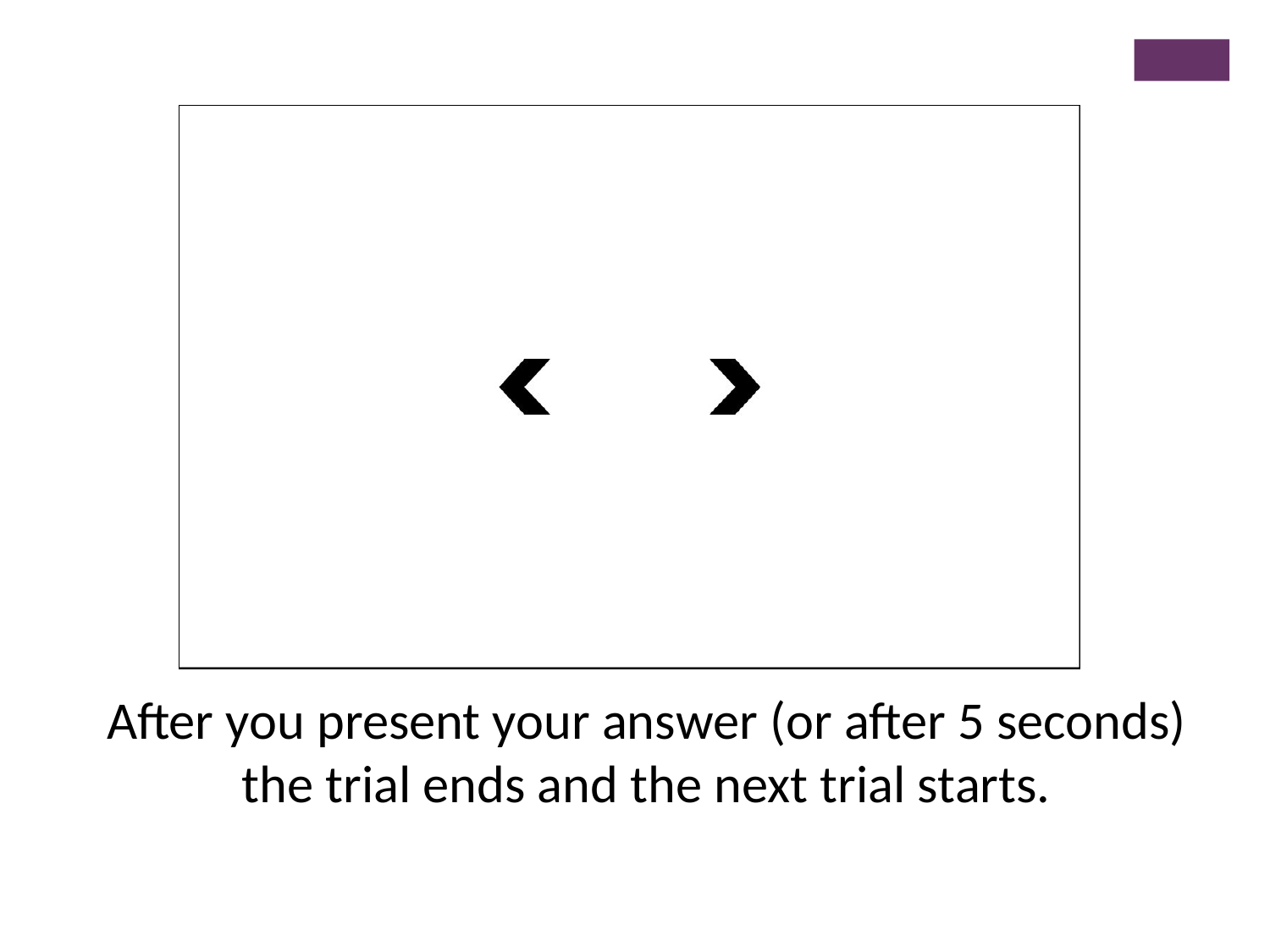

After you present your answer (or after 5 seconds) the trial ends and the next trial starts.

## Slide 11
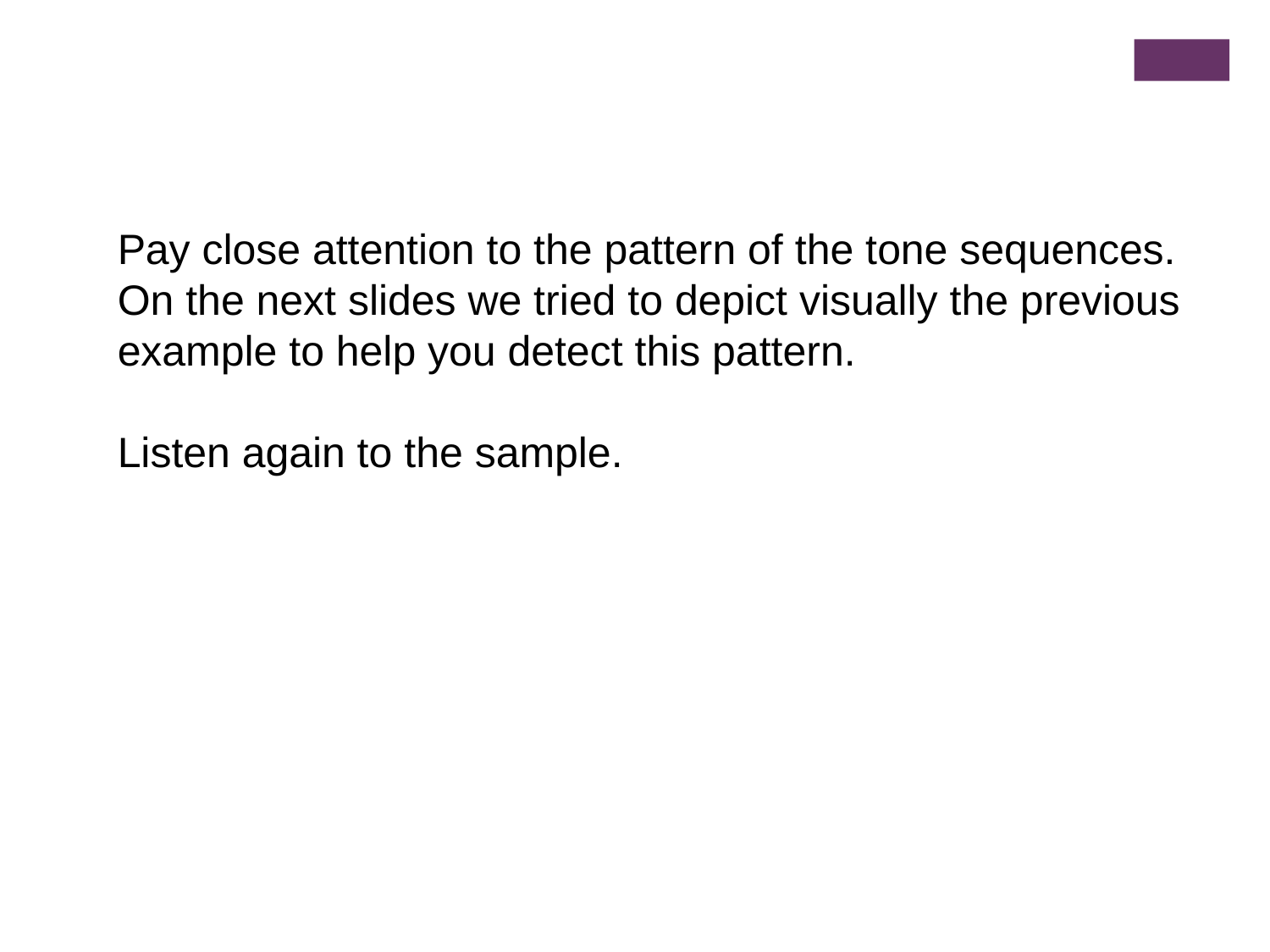

Pay close attention to the pattern of the tone sequences.
On the next slides we tried to depict visually the previous example to help you detect this pattern.
Listen again to the sample.

## Slide 12
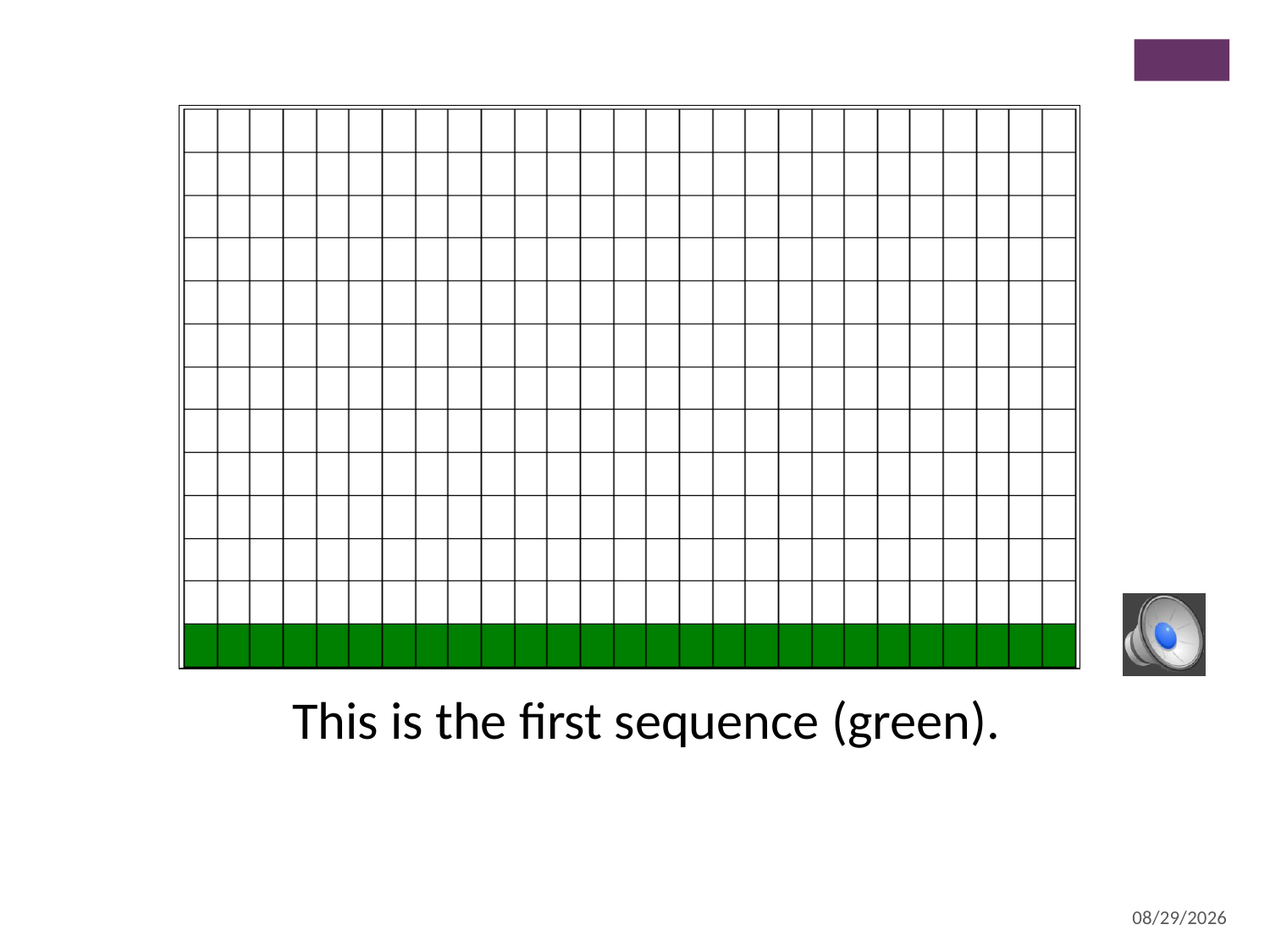

This is the first sequence (green).
11/25/13

## Slide 13
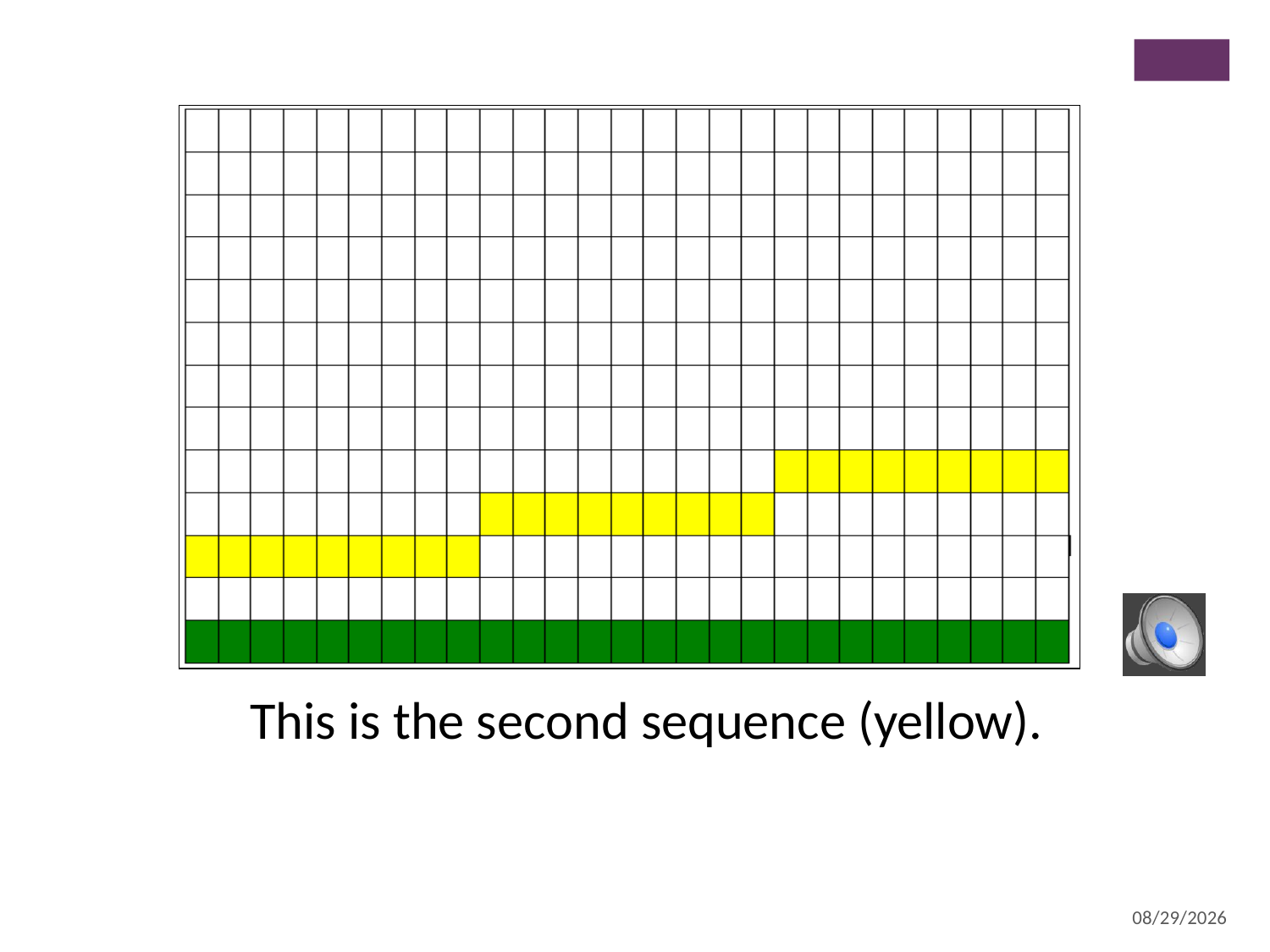

This is the second sequence (yellow).
11/25/13

## Slide 14
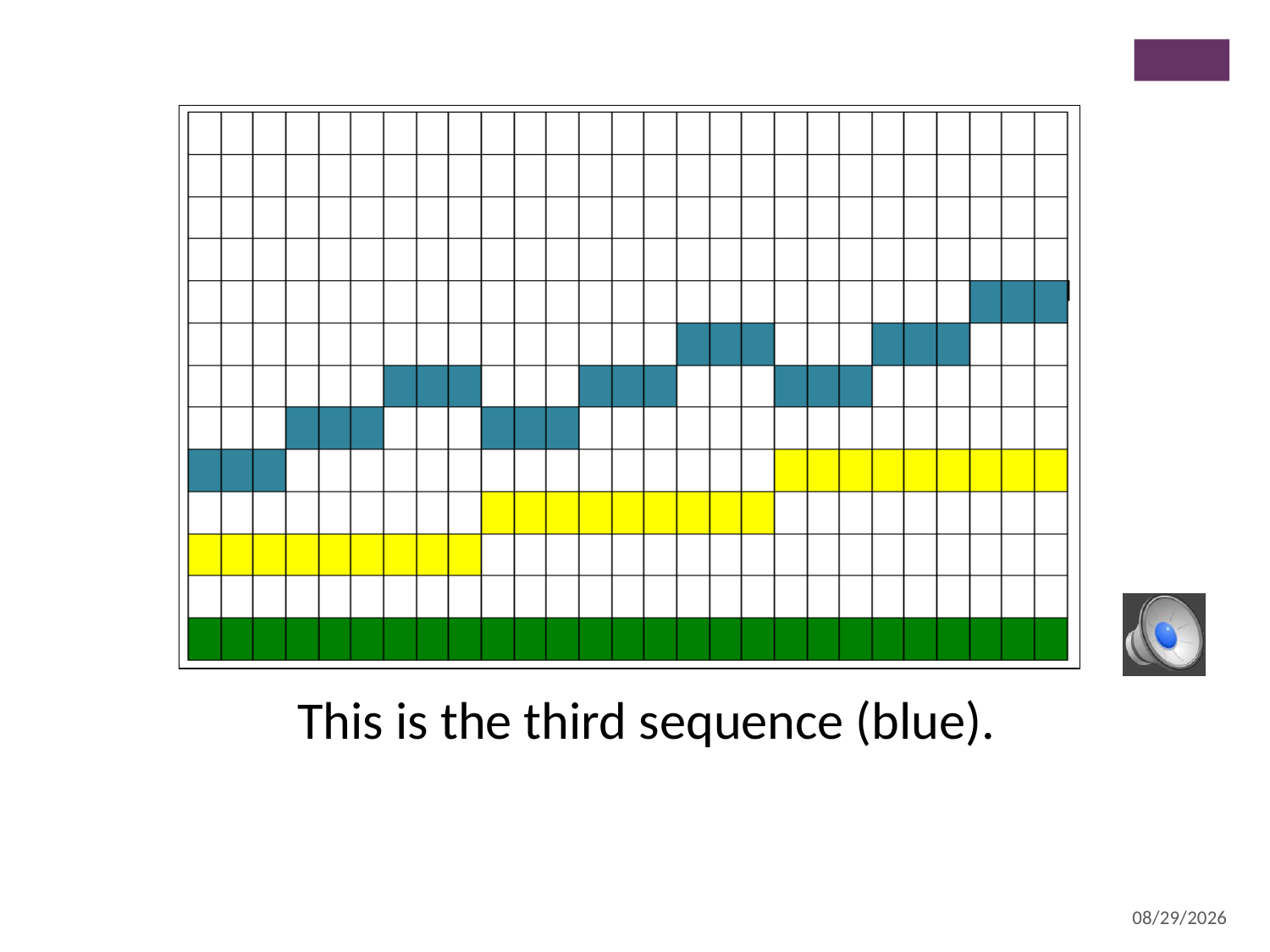

This is the third sequence (blue).
11/25/13

## Slide 15
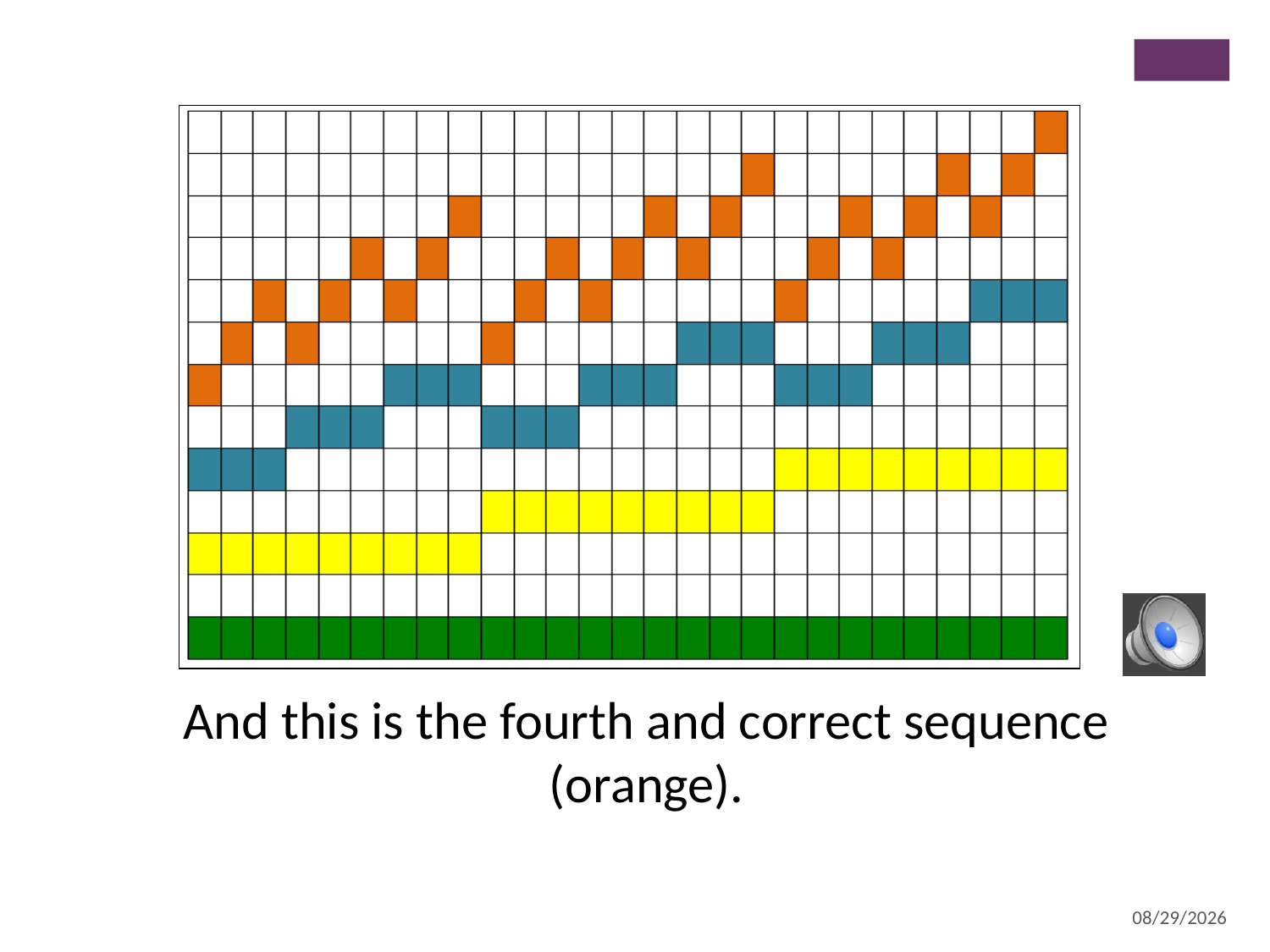

And this is the fourth and correct sequence (orange).
11/25/13

## Slide 16
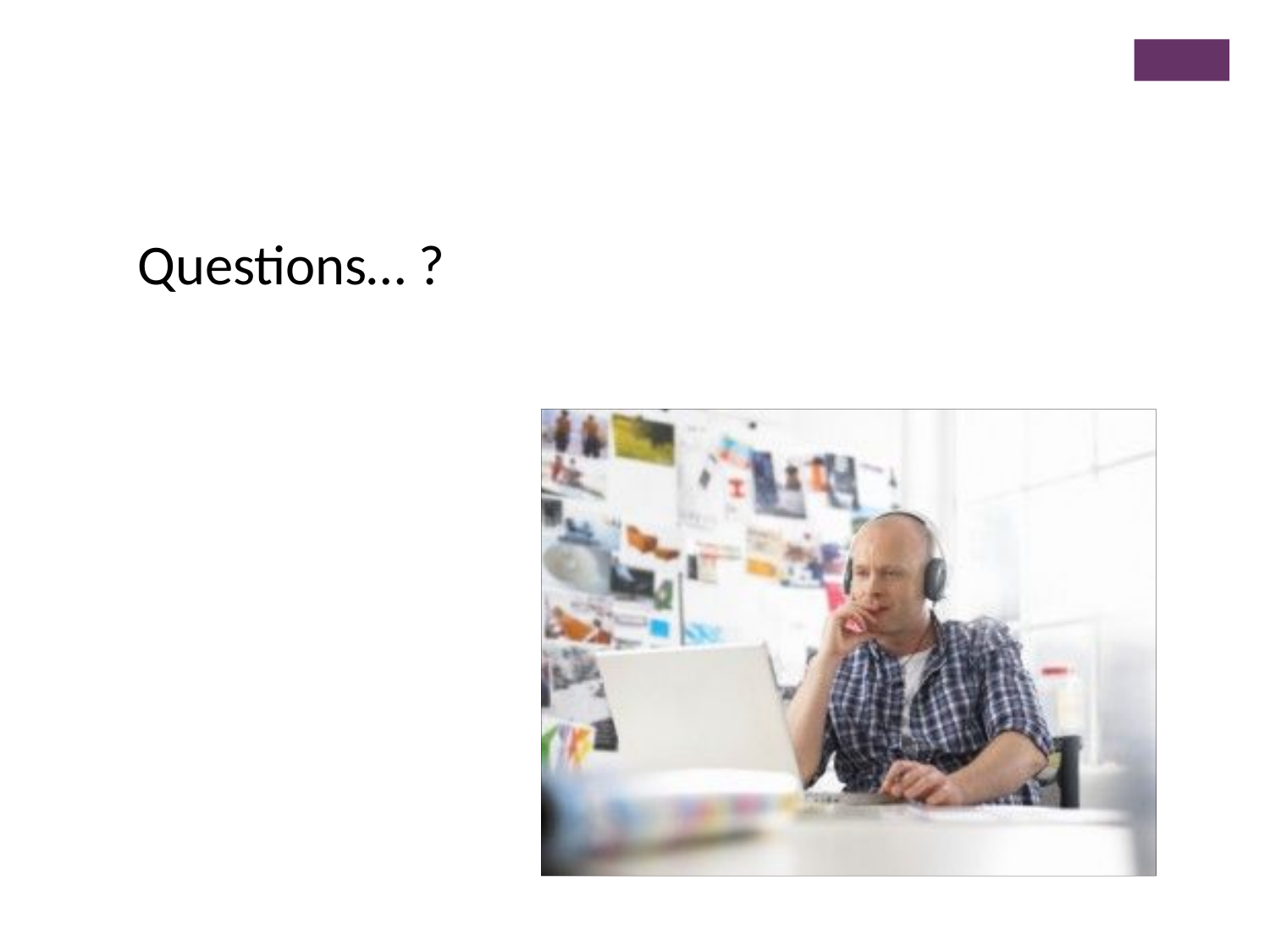

Questions… ?
