# Supplementary material for: Recursive music elucidates neural mechanisms supporting the generation and detection of melodic hierarchies
Source: Brain Struct Funct. 2020 Jun 26;225(7):1997–2015. doi: 10.1007/s00429-020-02105-7 (PMC7473971; doi:10.1007/s00429-020-02105-7)
Supplement: Supplementary file 2 — Supplementary file2 (DOCX 6717 kb) [file 429_2020_2105_MOESM2_ESM.docx]

**Supplementary Materials**

1. **Activations Full Test Sound Phase (complete 7.4 seconds)**


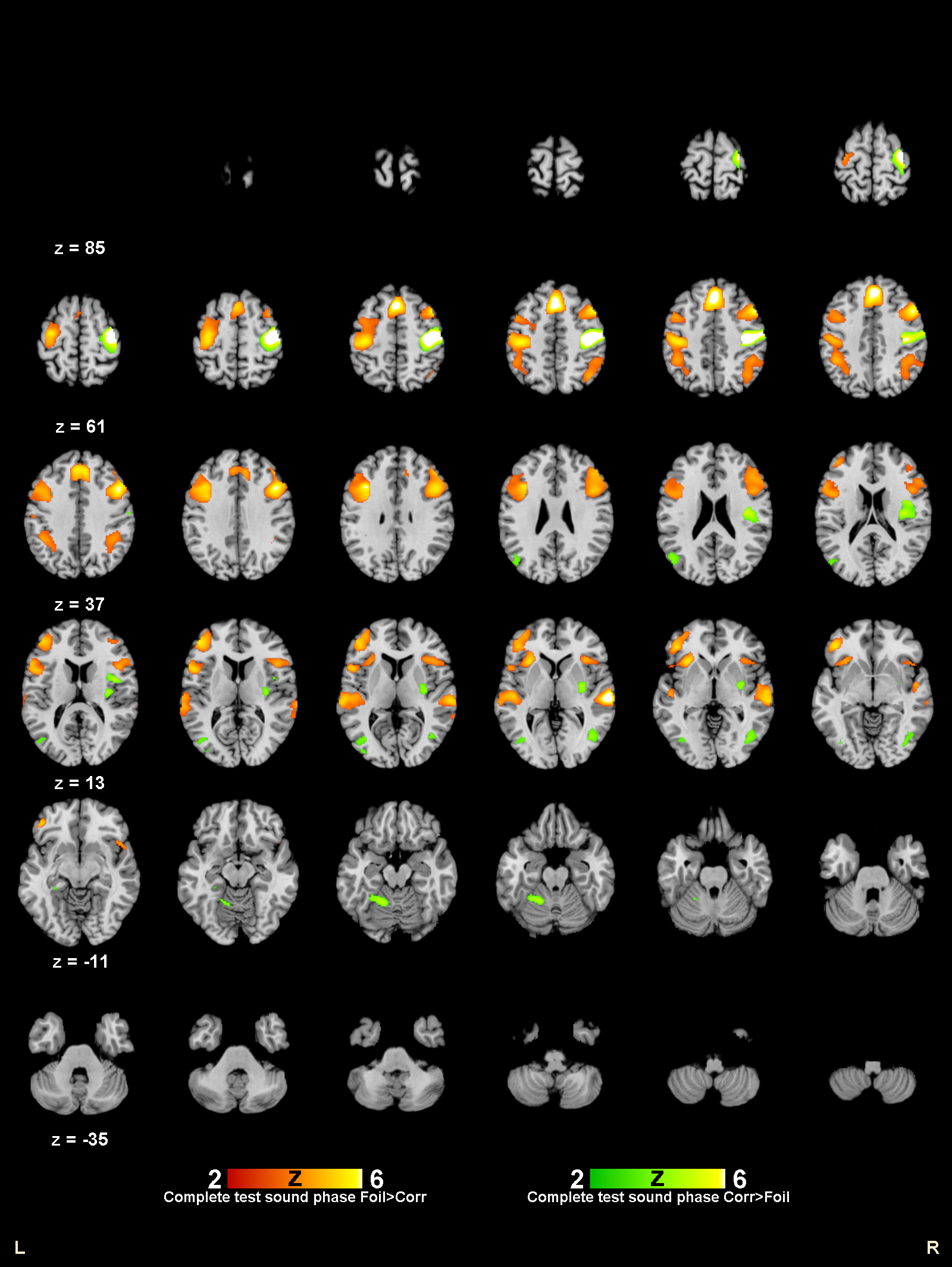


1. **Activity patterns during Generation phase**
   1. **Recursion > Iteration**

**
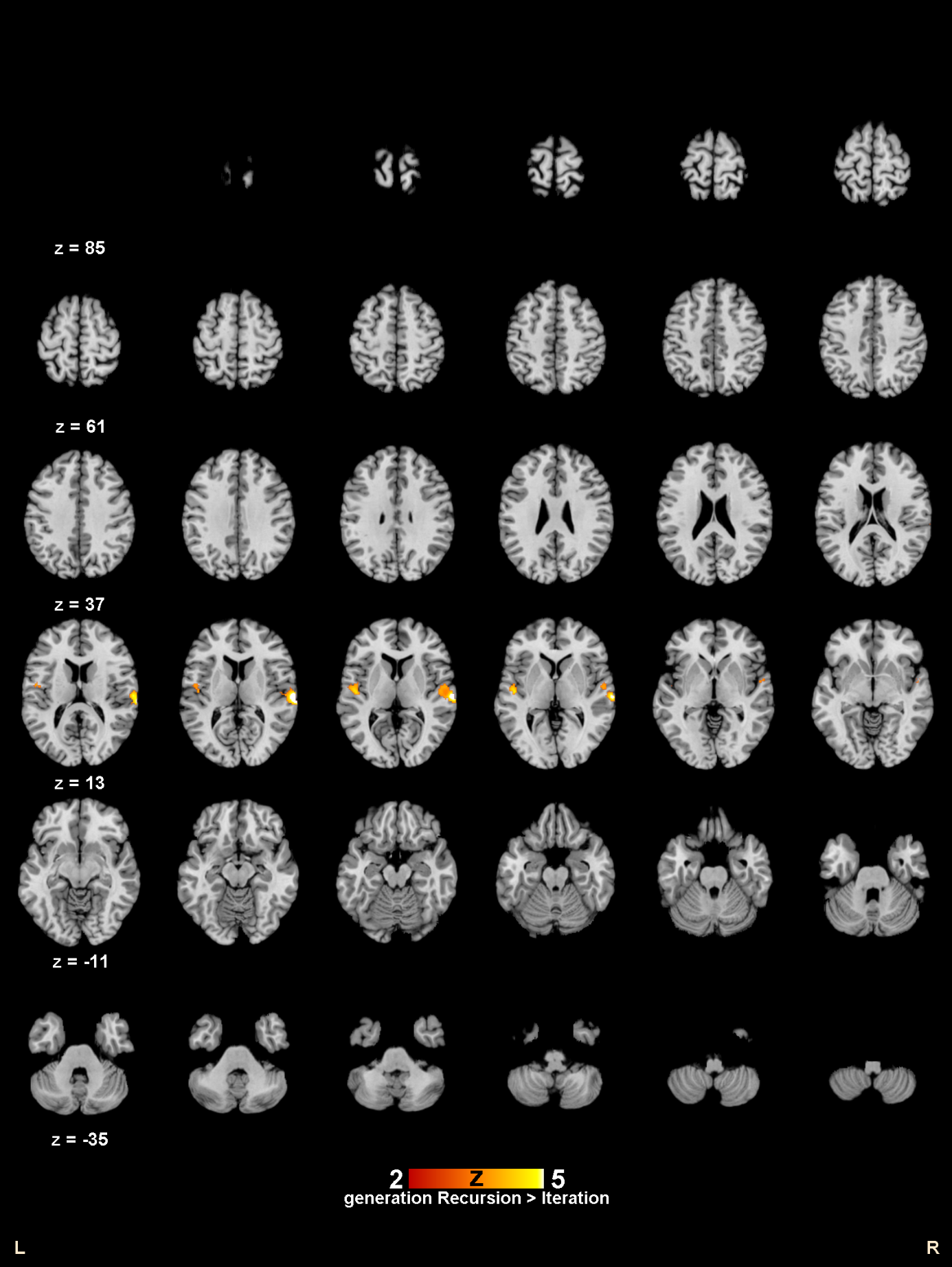
**

- 1. **Recursion > Repetition**

**
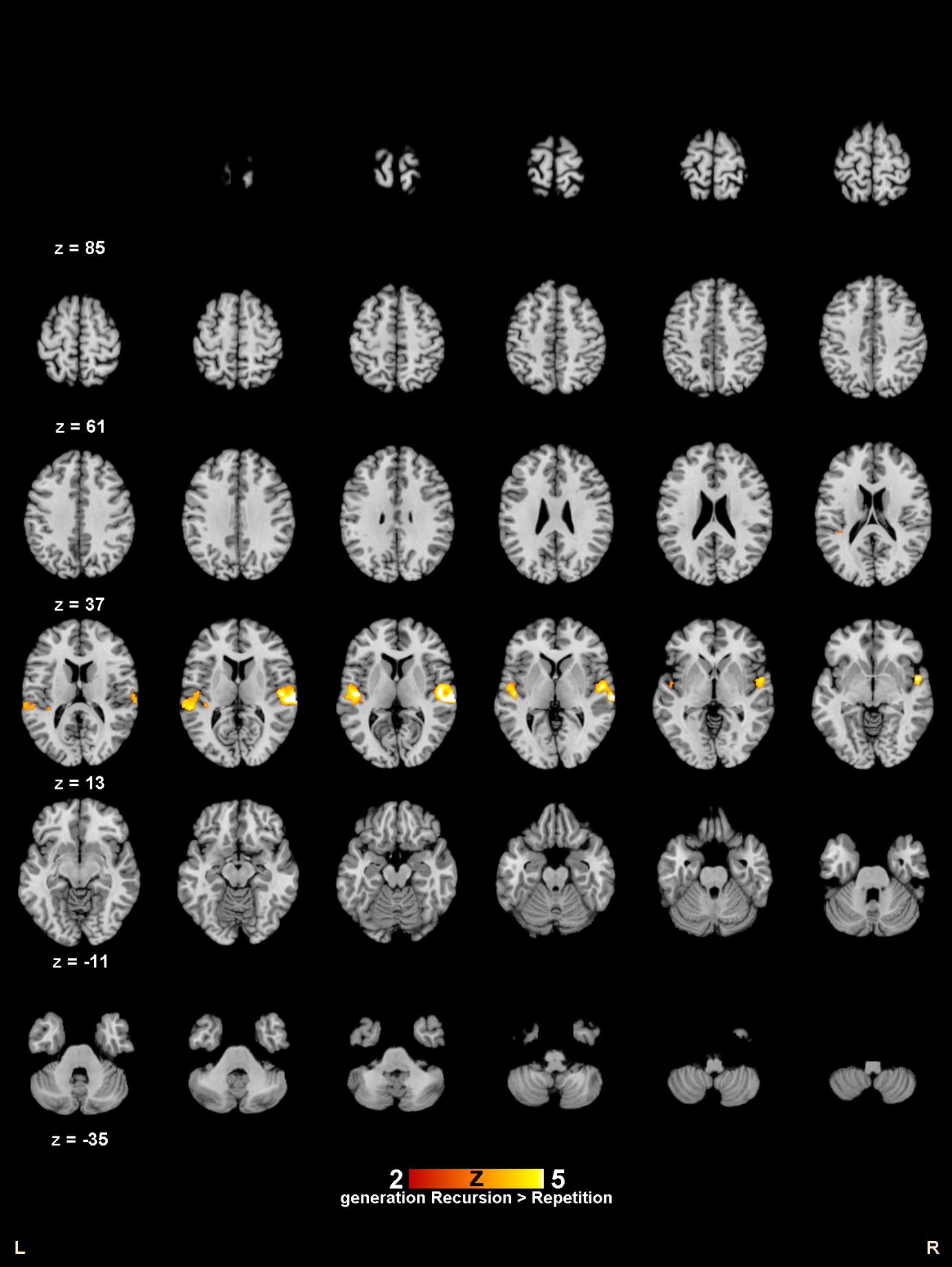
**

1. **Activity patterns during the steps I, II and III (i.e. trial period BEFORE planning)**
   1. **Recursion > Iteration**


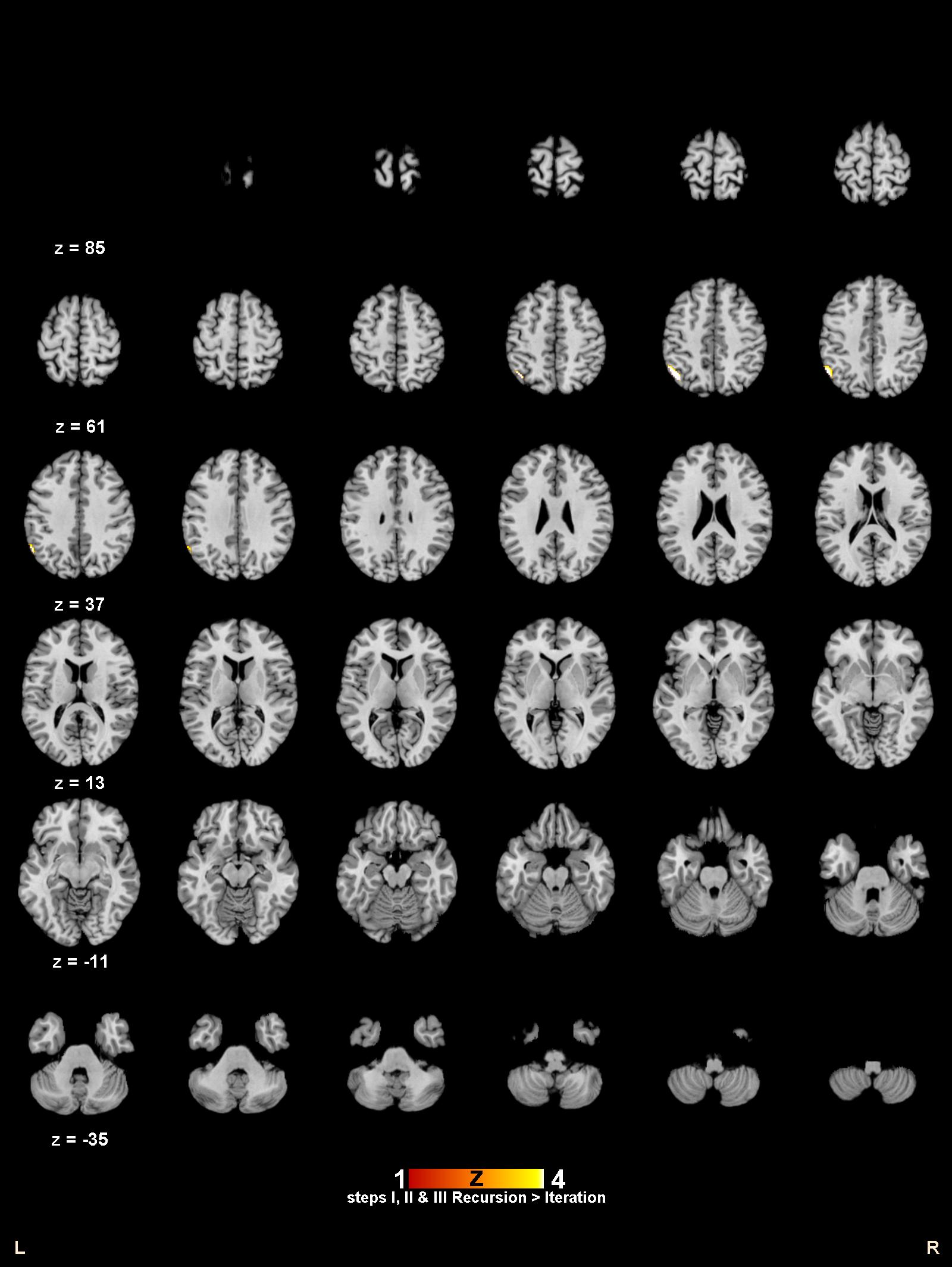


1. **Recursion > Repetition**


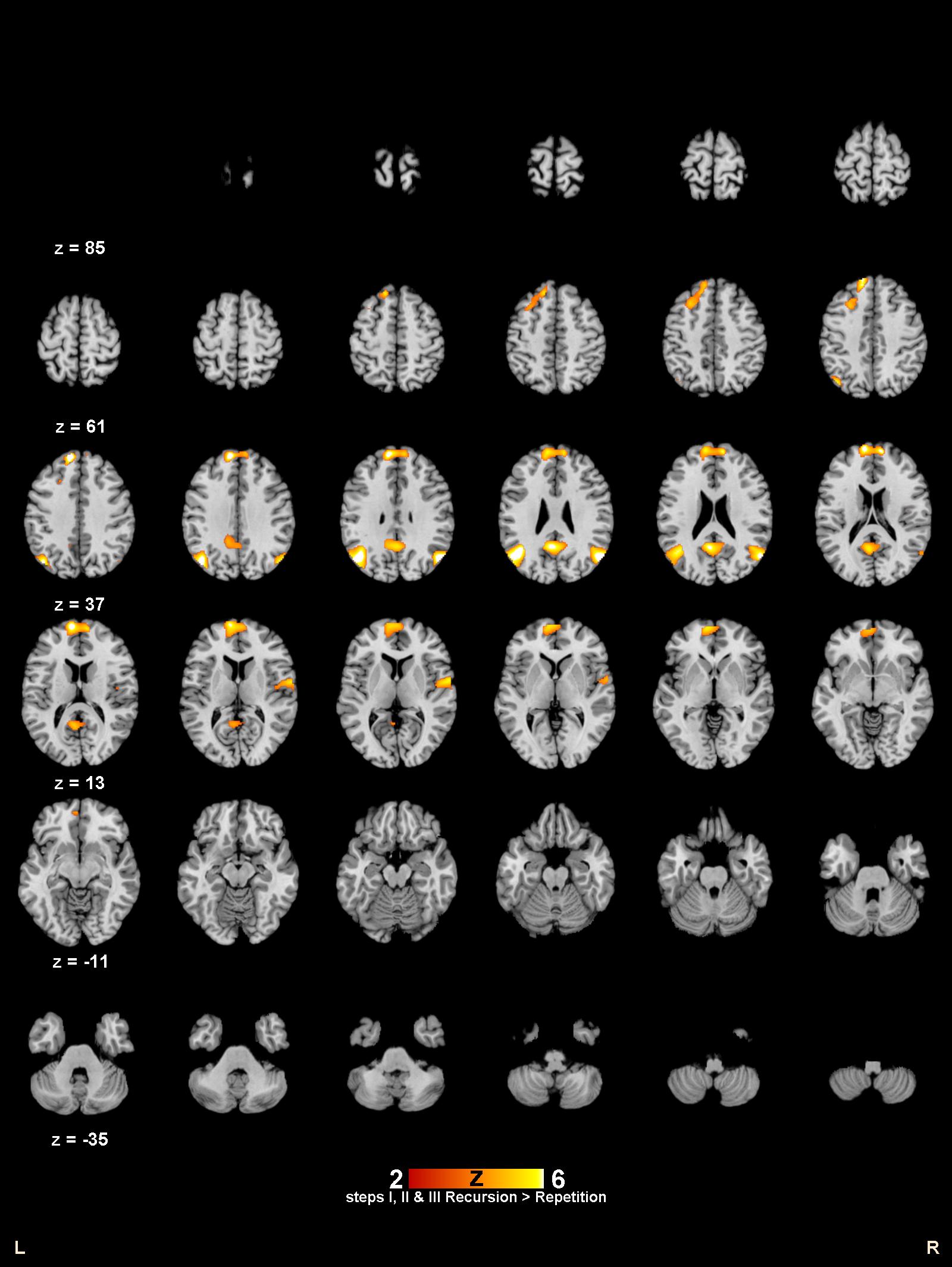


1. **Repetition > Recursion**

**
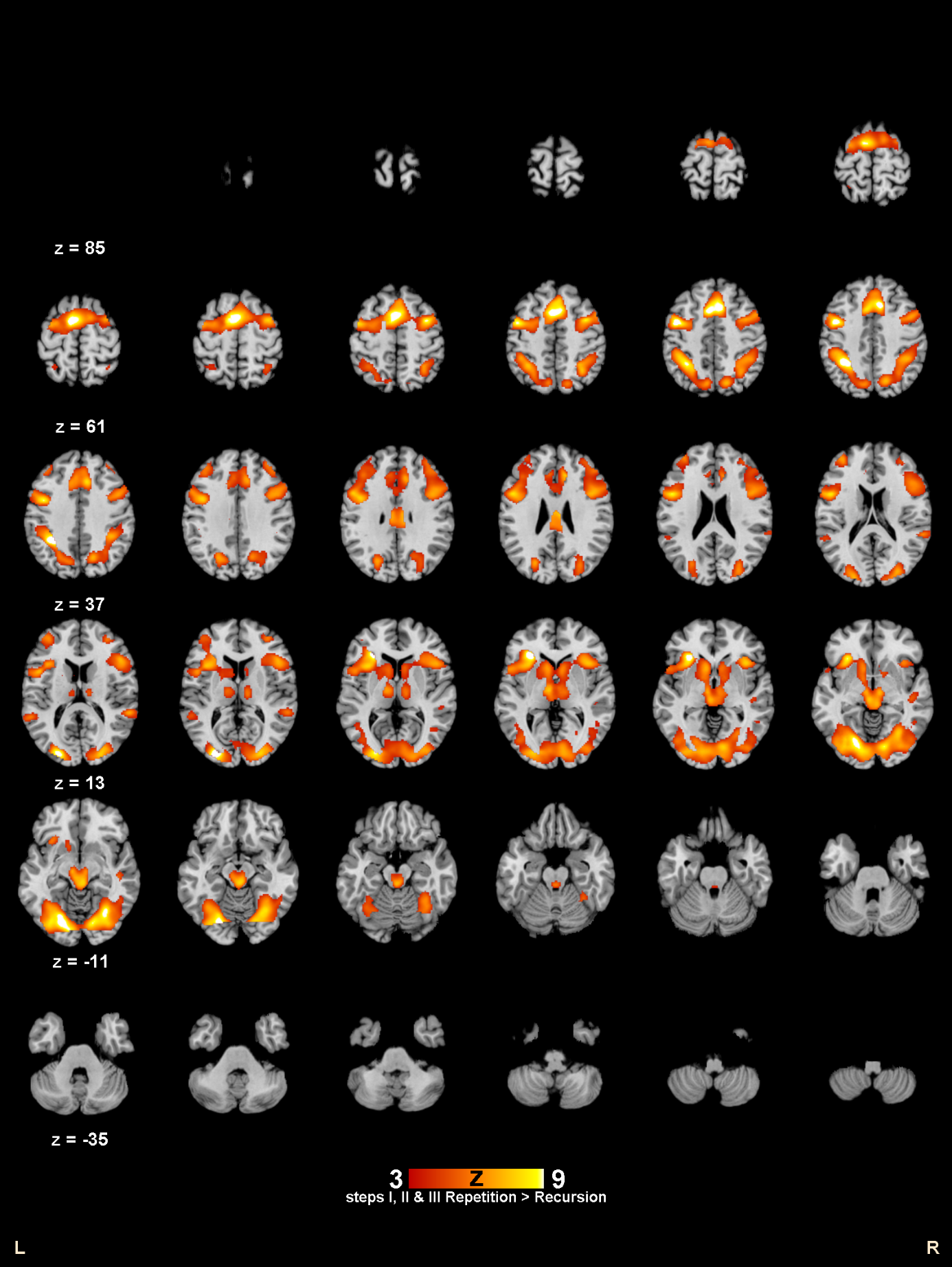
**

1. **Repetition > Iteration**

**
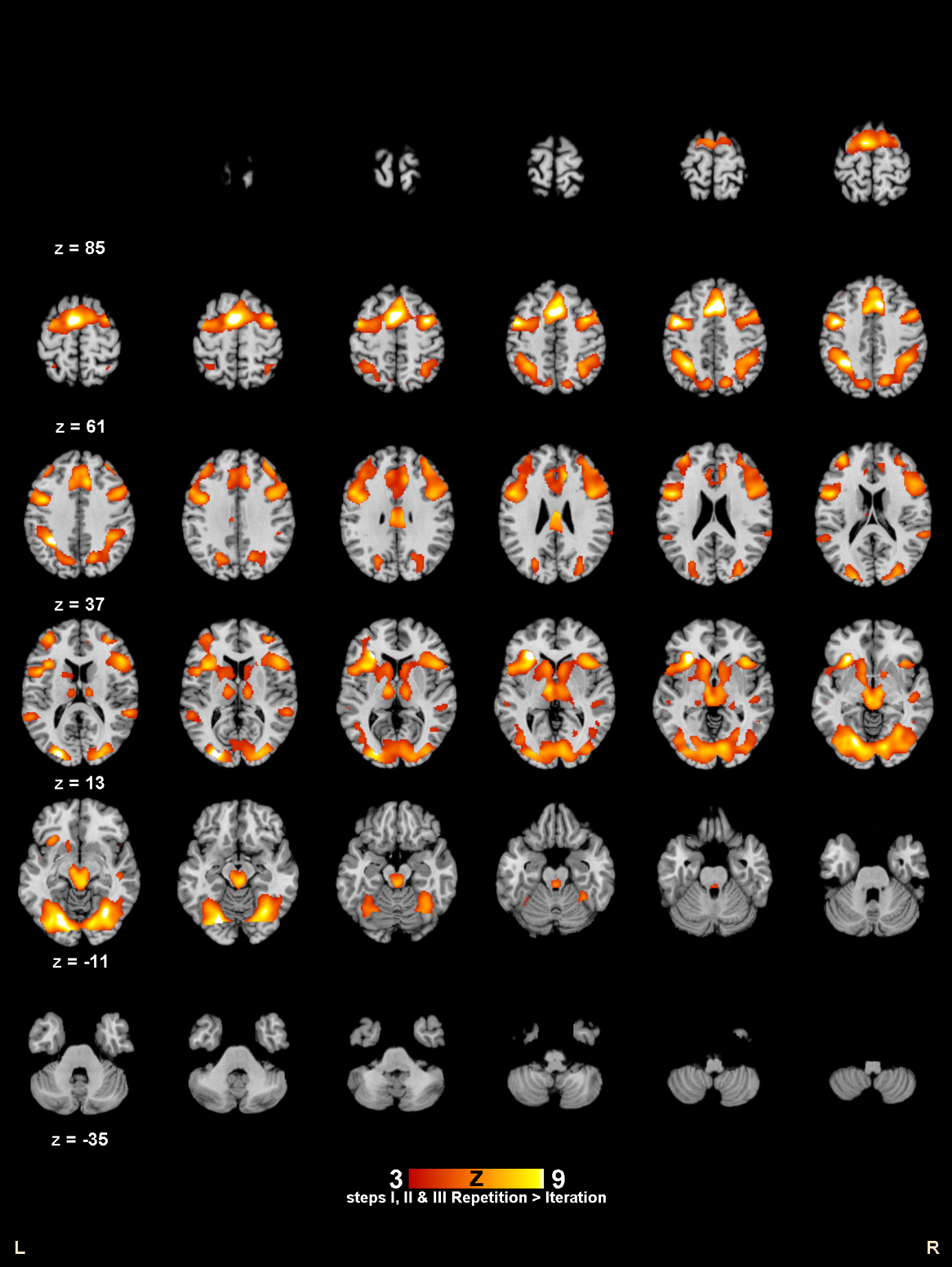
**
